# The PvNF-YA1 and PvNF-YB7 Subunits of the Heterotrimeric NF-Y Transcription Factor Influence Strain Preference in the *Phaseolus vulgaris–Rhizobium etli* Symbiosis

**DOI:** 10.3389/fpls.2019.00221

**Published:** 2019-02-28

**Authors:** Carolina Rípodas, Melisse Castaingts, Joaquín Clúa, Julieta Villafañe, Flavio Antonio Blanco, María Eugenia Zanetti

**Affiliations:** Instituto de Biotecnología y Biología Molecular, Facultad de Ciencias Exactas, Universidad Nacional de La Plata, La Plata – Centro Científico y Tecnológico La Plata, Consejo Nacional de Investigaciones Científicas y Técnicas, La Plata, Argentina

**Keywords:** nodulation, nitrogen fixation, Nuclear Factor Y, rhizobia, transcription factors

## Abstract

Transcription factors of the Nuclear Factor Y (NF-Y) family play essential functions in plant development and plasticity, including the formation of lateral root organs such as lateral root and symbiotic nodules. NF-Ys mediate transcriptional responses by acting as heterotrimers composed of three subunits, NF-YA, NF-YB, and NF-YC, which in plants are encoded by relatively large gene families. We have previously shown that, in the *Phaseolus vulgaris* × *Rhizobium etli* interaction, the PvNF-YC1 subunit is involved not only in the formation of symbiotic nodules, but also in the preference exhibited by the plant for rhizobial strains that are more efficient and competitive in nodule formation. PvNF-YC1 forms a heterotrimer with the PvNF-YA1 and PvNF-YB7 subunits. Here, we used promoter:reporter fusions to show that both *PvNF-YA1* and *PvNF-YB7* are expressed in symbiotic nodules. In addition, we report that knock-down of *PvNF-YA1* and its close paralog *PvNF-YA9* abolished nodule formation by either high or low efficient strains and arrested rhizobial infection. On the other hand, knock-down of *PvNF-YB7* only affected the symbiotic outcome of the high efficient interaction, suggesting that other symbiotic NF-YB subunits might be involved in the more general mechanisms of nodule formation. More important, we present functional evidence supporting that both PvNF-YA1 and PvNF-YB7 are part of the mechanisms that allow *P. vulgaris* plants to discriminate and select those bacterial strains that perform better in nodule formation, most likely by acting in the same heterotrimeric complex that PvNF-YC1.

## Introduction

Nitrogen (N) is an essential macronutrient for plant growth and development since it is part of many biological molecules such as nucleic acids, proteins, vitamins, and chlorophyll (Wang et al., 2012); however, its availability is frequently limited in soils of both natural and agronomical ecosystems. Most legume species overcome N limitation by establishing a symbiosis with N-fixing bacteria from different genera known as rhizobia. This interaction results in the formation of a new postembryonic root organ, the nodule, where bacteria allocate and convert atmospheric N to reduced forms that will be incorporated into the plant metabolism. The root nodule symbiosis (RNS) can be divided into three stages. In the pre-symbiotic stage, the two organisms recognize each other through an exchange of diffusible molecules. Under N limiting conditions, roots of legumes exudate flavonoids/isoflavonoids that are perceived by rhizobia (Weston and Mathesius, 2013), which activate the synthesis and secretion of key molecules called Nod factors (NFs; Denarie et al., 1996). NFs are perceived by the host plant inducing the first morphological response, the curling of the root hair around the bacterial microcolony, which results in the formation of an infection pocket (Lerouge et al., 1990). The second stage is the infection, which consists in the penetration of bacteria into host tissues through a tubular structure referred to as the infection thread (IT). Concomitantly with this infection process, cell divisions are initiated in the root cortex of the host to form the nodular primordium. The third and last stage consists in the development of the nodule and the release of bacteria from the ITs into the host cells to form organelle-like structures called symbiosomes (Popp and Ott, 2011), where biological N fixation will take place.

Morphological and developmental responses observed during RNS are initiated by the binding of NFs to LysM domain-containing receptors located at the plant plasma membrane, which results in the hierarchical activation of a set of transcriptions factors (TFs). Specific members of the Nuclear Factor Y (NF-Y) gene family of TFs have been implicated at different stages of the RNS, from epidermal infection to nodule development. NF-Ys are evolutionary conserved heterotrimeric TFs composed of three subunits (NF-YA, NF-YB, and NF-YC). Genes encoding NF-Y subunits have diversified in the plant lineage forming relatively large gene families with specific functions (Petroni et al., 2012; Laloum et al., 2013). *In Medicago truncatula*, two NF-YA subunits, *MtNF-YA1* and *MtNF-YA2*, play important roles not only at early stages of rhizobial infection, but also at later stages of the RNS mediating both nodule organogenesis and the persistence of nodule meristems (Combier et al., 2006, 2008; Laloum et al., 2014; Laporte et al., 2014). On the other hand, *M. truncatula MtNF-YC1* and *MtNF-YC2* genes are required for nodule organogenesis, but not for intracellular infection by rhizobia (Baudin et al., 2015). Two NF-Y subunits of *Lotus japonicus*, *LjNF-YA1* and *LjNF-YB1*, were identified as direct transcriptional targets of the master symbiotic regulator Nodule Inception (NIN). Knock-down of *LjNF-YA1* by RNA interference (RNAi) arrested cell divisions associated with nodule formation, but did not affect epidermal infection. On the other hand, overexpression of *LjNF-YA1* stimulates cell proliferation, a phenotype that was enhanced by co-expression of *LjNF-YB1* (Soyano et al., 2013). In common bean (*Phaseolus vulgaris*), *PvNF-YC1* was identified as a key TF required for both nodule organogenesis and infection by *Rhizobium etli*, the predominant species present in common bean nodules (Zanetti et al., 2010).

*Phaseolus vulgaris* originated in Mesoamerica and further expanded to South America, resulting into two gene pools at distinct centers of genetic diversification (CGDs): the Mesoamerican and the Southern Andes CGDs (Bitocchi et al., 2012). These gene pools have undergone parallel and independent domestication at each CGD, thus the characteristics of each gene pool are evident in both wild and domesticated accessions (Bitocchi et al., 2013). The abundance of *R. etli* strains in each CGD has been correlated with a polymorphism of the *nodC* gene of *R. etli*, which encodes an *N*-acetylglucosamine transferase involved in the first steps of NF synthesis. Strains bearing the *nodC*-α allele (hereafter *nodC*-α strains) are predominant in Mesoamerican soils, whereas those carrying the *nodC*-δ allele are highly represented in the Andean region (Aguilar et al., 2004). Wild and domesticated beans from each CGD are more efficiently nodulated by those strains that are more abundant in the soils of the cognate geographical region (Peltzer Meschini et al., 2008). More interesting, nodules of Mesoamerican beans co-inoculated with an equicellular mixture of both rhizobial strains were predominantly occupied by *nodC*-α strains (Aguilar et al., 2004), leading to the suggestion that Mesoamerican beans have developed molecular mechanisms that allow them to discriminate and select those strains that have coevolved in the same CGD. A key component of this mechanism is the above-mentioned *PvNF-YC1* subunit of the NF-Y family of TFs (Zanetti et al., 2010). Overexpression of *PvNF-YC1* in Mesoamerican beans was sufficient not only to improve the symbiotic outcome (i.e., nodule number and shoot dry weight) of the less efficient strains carrying the *nodC*-δ allele, but also to alter nodule occupancy by *nodC*-α and *nodC*-δ strains, exposing that competitions in the rhizosphere can be controlled by the plant (Zanetti et al., 2010). Since NF-Ys act as heterotrimers to promote transcriptional activation/repression, we sought to identify and characterize the NF-YA and NF-YB subunits that form the symbiotic functional heterocomplex. Given the symbiotic specific expression pattern exhibited by *PvNF-YA1*, *PvNF-YA9*, and *PvNF-YB7* genes (Ripodas et al., 2015) and the physical interaction of PvNF-YC1 with PvNF-YA1 and PvNF-YB7 subunits (Baudin et al., 2015), we selected these members to conduct a functional characterization of their role in the RNS and in the strain preference observed in Mesoamerican beans. Here, we describe that simultaneous silencing of *PvNF-YA1* and its closest homolog, *PvNF-YA9*, impaired nodule organogenesis triggered by either *nodC-α* or *nodC-*δ strains and reduced bacterial infection. Interestingly, overexpression of PvNF-YA1, but not PvNF-YA9, was sufficient to alter nodule occupancy in roots co-inoculated with *nodC-α* and *nodC-*δ strains. On the other hand, knock-down of *PvFN-YB7* affected the number of nodules developed by a *nodC-α*, but not a *nodC-δ* strain, and altered nodule occupancy. All together, the results presented here highlight the functional implication of the heterotrimer formed by PvNF-YA1, PvNF-YB7 and PvNF-YC1 not only in the establishment of the RNS, but also in the mechanisms that determine strain specificity within the *P. vulgaris* ×*R. etli* interaction.

## Materials and Methods

### Biological Material and Generation of Composite Plants by *Agrobacterium rhizogenes* Transformation

Plant growth and transformation were performed essentially as previously described (Blanco et al., 2009; Zanetti et al., 2010). Briefly, *P. vulgaris* seeds were surface sterilized and germinated on 10% (w/v) agar-H_2_O for 2 days. Seedling were transferred to pots containing vermiculite and watered with Fahraeus media supplemented with 8 Mm KNO_3_. Five days after transplantation, *P. vulgaris* plants were inoculated in the stem with a saturated suspension of *Agrobacterium rhizogenes* strain K599 using a syringe. Approximately 10 days after transformation, when hairy roots have emerged from the inoculation sites, the main root system was removed by cutting the stem 1 cm below the site of inoculation. Composite plants consisting on a wild type aerial part and transgenic hairy roots were transferred to acrylic boxes containing agar-Fahraeus covered with paper. Alternatively, for co-inoculation experiments, composite plants were transferred to pots containing vermiculite and watered with Fahraeus media. *R. etli* strains SC15 (*nodC*-α) and 55N1 (*nodC*-δ) were previously reported (Aguilar et al., 2004). The *R. etli* strain CFNx5 (*nodC*-α) expressing the DsRed protein was previously generated and described (Battaglia et al., 2014).

### Vector Construction

To generate localization and/or overexpression constructs, the open reading frame of PvNF-YA1 was amplified by PCR using primers PvNF-YA1 OE F, PvNF-YA1 OE R (Supplementary Table S1), and cloned into the pENTR/D-TOPO vector (Invitrogen), creating pENTR-NF-YA1. For overexpression, the pENTR-NF-YA1 was recombined into the destination vector p35S:HF-GATA (Mustroph et al., 2009). Later on, the FLAG-PvNF-YA1 fragment was amplified by PCR using specific primers and cloned into the pENTR/D-TOPO to create pENTR-FLAG-NF-YA1, which was subsequently recombined into the final destination vector pK7WG2D, which carries EgfpER as a screenable marker for early visualization and selection of the transgenic roots (Karimi et al., 2002). For subcellular localization, a translational fusion of PvNF-YA1 to the C-terminal end of GFP was generated by recombination of the pENTR-NF-YA1 with pMDC43 (Curtis and Grossniklaus, 2003). For the overexpression of PvNF-YA9, the ORF of this transcript was synthetized by Life Technologies to be used as level-0 module in GoldenGate cloning^1^. Assembled level-1 modules expressing the fusion FLAG-PvNF-YA9 under the control of the constitutive promoter CaMV35S and a pAtUbi:GFP fusion as a transgenic root selection marker were finally cloned in a level-2 binary vector backbone EC50505^1^. For histochemical assays to measure glucuronidase activity, a 1.5 kb fragment of the promoter sequences of *PvNF-YA1* and *PvNF-YB7* was amplified with specific primers from common bean genomic DNA, cloned in the pENTR/D-TOPO vector, and finally introduced by recombination into the destination vector pKGWFS7, driving the expression of the fusion GFP-GUS. For silencing of *PvNF-YA1/A9* by RNAi, a 217 bp fragment corresponding to the 3^′^ UTR of *PvNF-YA1* was amplified by PCR, using PvNF-YA1/A9 RNAi F and PvNF-YA1/A9 RNAi R primers (Supplementary Table S1) and *P. vulgaris* cDNA as a template. Similarly, a fragment corresponding to the 3^′^ UTR was amplified using gene specific primers for knock-down of *PvNF-YB7* (Supplementary Table S1). Each PCR product was cloned into the entry vector pENTR/D-TOPO and recombined into the destination vector pK7GWIWG2D (II) (Karimi et al., 2002) to finally produce *PvNF-YA1/A9* RNAi and *PvNF-YB7* RNAi constructs, respectively. All binary vectors were introduced into *Agrobacterium tumefaciens* GV3101 and/or *A. rhizogenes* K599 by electroporation and then used for agroinfiltration of *Nicotiana benthamiana* leaves or for the generation of transgenic hairy roots in *P. vulgaris*.

### β-Glucuronidase Activity

Activity of the enzyme β-glucuronidase was determined in *P. vulgaris* roots and nodules formed at 7 and 14 dpi with strain CFNX5, a *R. etli nodC-*α strain that expresses the fluorescent protein Ds-Red. Roots were cut into 2 cm sections and then infiltrated with a solution of the dye reactive: 100 mM TRIS-HCl pH 7, 2 mM X-Gluc (5-bromo-4-cloro-3-indolil-β-D-glucoronic acid, 0.01% v/v Triton X-100, 50 mM NaCl, 2.0 mM potassium ferrocyanide). Tissue was incubated at 37°C for 1–10 h until color development. After incubation, roots were observed by bright field microscopy to visualize GUS staining in whole roots or, in some cases, selected roots and nodules were embedded in 4% (w/v) agarose and cut to thin sections (55 μm) using a Leica VT1000 S Vibrating blade microtome. Tissue sections were analyzed by bright field microscopy in an inverted microscope (OLYMPUS IX51).

### Phenotypic Analysis

Composite plants were generated and inoculated as described (Peltzer Meschini et al., 2008; Blanco et al., 2009). Primary and lateral root length and density were measured as previously reported (Battaglia et al., 2014). Nodule quantification and dry weight determination were performed as previously described (Zanetti et al., 2010). Non-transgenic (not fluorescent roots) were excised from the root system before phenotypic analysis before inoculation with rhizobia, thus only nodules formed in transgenic roots were taken into account. IT quantification and classification was performed essentially as previously described by Battaglia et al. (2014). Briefly, composite plants were inoculated with a *R. etli* strain CFNx5 that constitutively expresses the fluorescent protein Ds-Red. ITs were visualized and quantified under UV light. For co-inoculation experiments, composite plants were transferred to pots containing vermiculite. Five days after transplantation, roots were inoculated with 10 ml of a mixture of *R. etli* strains SC15 and 55N1 (ratio 1:1) as previously described (Zanetti et al., 2010). Four weeks after co-inoculation, more than 100 nodules from 10 independent plants for each construct were excised, crushed, and plated in Congo Red-YEM agar plates. The color of the bacteria grown on this media was recorded. Bacterial DNA was extracted, subjected to PCR amplification of the *nodC* gene, and digested with *Hinf*I to determine the restriction profile of the *nod*C gene as reported (Aguilar et al., 2004). All experiments were conducted in three independent biological replicates.

### Subcellular Localization Assays

The PvNF-YA1-GFP construct was introduced in *A. tumefaciens* GV3101 and *A. rhizogenes* K599 by electroporation. *A. tumefaciens* carrying PvNF-YA1 was co-infiltrated into *N. benthamiana* leaves with the viral silencing inhibitor protein P19 (Voinnet et al., 2003) as described (Battaglia et al., 2014). The subcellular localization in common beans roots was done by generation of *P. vulgaris* composite plants expressing the PvNF-YA1-GFP fusion (Zanetti et al., 2010).

### RNA Extraction and Quantitative RT-PCR

RNA extraction, cDNA synthesis, and RT-qPCR assays in common bean were performed as previously reported (Ripodas et al., 2013). Transcript levels for each of the target genes were normalized to the endogenous elongation factor 1 α (*EF1α*) transcript levels. Primer sequences for quantitative RT-PCR analyses are shown in Supplementary Table S1. Data shown are mean values obtained in three or four independent biological experiments with two or three technical repeats.

### Microscopy and Imaging

Bright-field and epifluorescence imaging of ITs formation were performed as described (Battaglia et al., 2014). Confocal microscopy of *P. vulgaris* roots, *N. benthamiana* leaves, and ITs observation were made with a Leica confocal microscope (SP5) using 20X and 40X objectives. Samples were excited with argon laser and emission spectra used to detect the fluorescence were: GFP (498–550 nm) and DsRed (578–626 nm).

### Western Blots

Proteins from transgenic root tissue of individual composite plants were extracted, separated into 12% SDS-PAGE, and subjected to immunoblot analysis using anti-FLAG antibody (1:500; Sigma–Aldrich) as previously described (Zanetti et al., 2010).

## Results

### *PvNF-YA1* Is a Nuclear Localized TF Expressed in the Central Tissue of *P. vulgaris* Nodules

**FIGURE 1:**
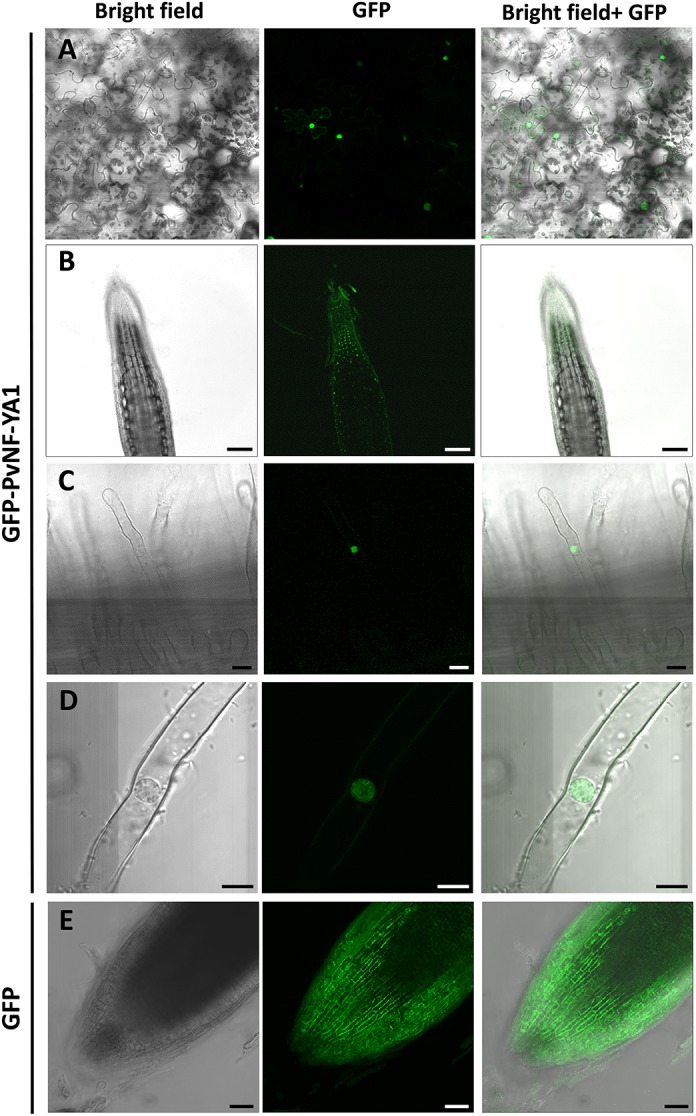
Subcellular localization of PvNF-YA1 in *N. benthamiana* leaves and *P. vulgaris* roots. **(A)** Subcellular localization of the translational fusion GFP-NF-YA1 in epidermal cells of leaves of *N. benthamiana*. Images obtained under white (bright field) and UV light (GFP) by confocal microscopy, as well as the merged image (bright field + GFP), are shown. Scale bar: 50 μm. *P. vulgaris* roots expressing the GFP-NF-YA1 fusion **(B–D)** or free GFP **(E)** were observed by confocal microscopy. Images show the tip of a lateral roots **(B,E)** or root hairs **(C,D)**. Bright field, GFP channel, and merged (bright field + GFP) images are shown. The integration in *Z*-axis of 15 confocal sections is shown in **E**. Scale bars: 100 **(B)**, 25 **(C)**, 8 **(D)**, and 50 μm **(E)**.

The current model for assembling of the heterotrimeric NF-Y complex proposes that the NF-YB interacts with NF-YC in the cytoplasm and the heterodimer translocates to the nucleus where it joins the NF-YA subunit. Consistent with this model, nuclear subcellular localization was described for several NF-YA proteins in different metazoan and plants species (Liu and Howell, 2010; Hackenberg et al., 2012; Baudin et al., 2015). In this study, nuclear subcellular localization of PvNF-YA1 was verified by expression of a translational fusion of PvNF-YA1 to the green fluorescent protein (GFP) into *N. benthamiana* leaf epidermal cells (Figure 1A). The GFP-PvNF-YA1 fusion protein was also detected in the nucleus of *P. vulgaris* root cells generated by *A. rhizogenes*–mediated transformation (Figure 1B), including epidermal root hairs (Figure 1C,D). As expected, free GFP was dispersed between the nucleus and the cytoplasm (Figure 1E).

**FIGURE 2:**
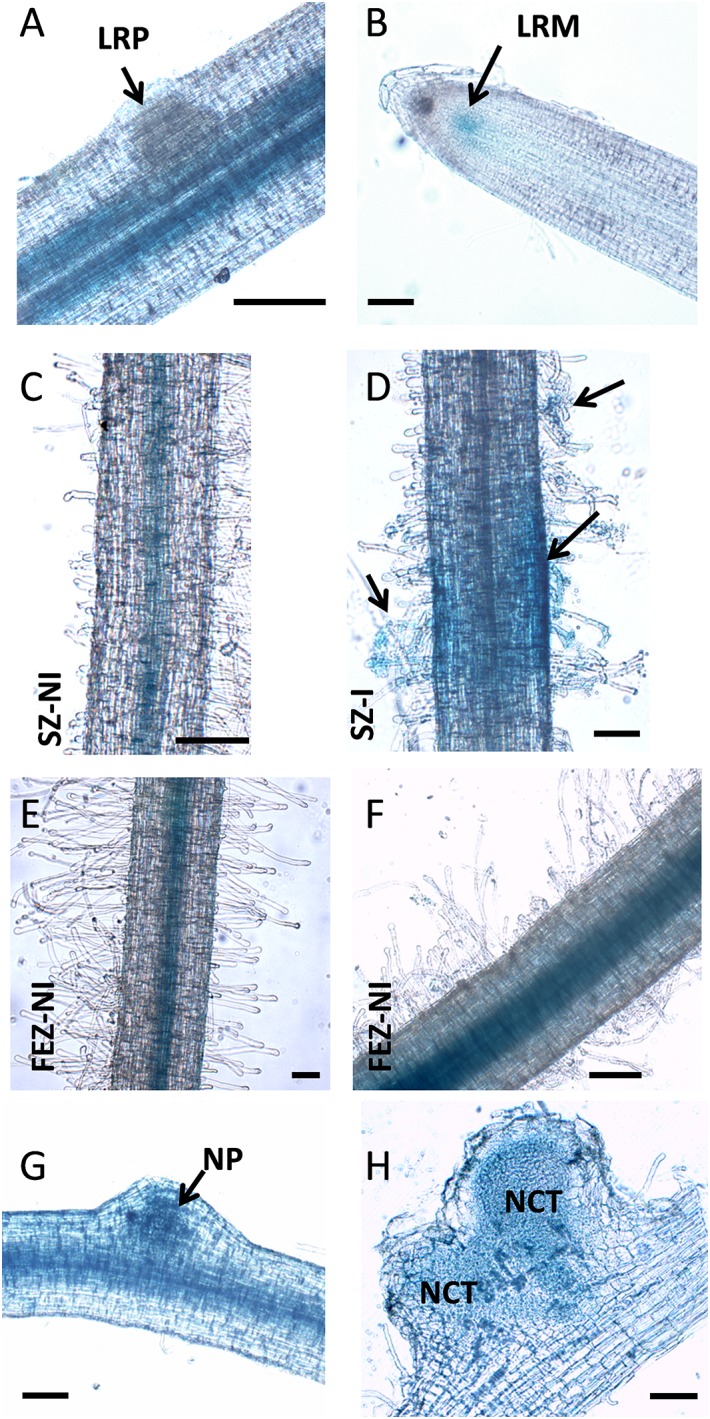
*PvNF-YA1* promoter is active in roots and symbiotic nodules of *P. vulgaris*. Histochemical GUS staining of roots and nodule transformed with the *ProPvNF-YA1:GFPGUS* construct. **(A)** Non-inoculated (NI) primary root which containing a lateral root primordia (LRP). **(B)** Lateral root meristematic (LRM) zone of a NI lateral root. **(C)** Susceptible zone (SZ) of a NI lateral root. **(D)** SZ of lateral root inoculated (I) with *R. etli* strain SC15. Arrows point to infection sites. **(E)** Zone of fully elongated root hairs (FEZ) on a NI lateral root. **(F)** FEZ of a lateral root inoculated with *R. etli* strain SC15. **(G)** Nodule primordia (NP) or **(H)** nodules developed by *R. etli* strain SC15 at 6 or 14 dpi. NCT: nodule central tissue. Pictures are representative of images observed in more than three independent experiments. Bars: 200 μm.

Previous expression analysis of NF-YA family members reveled that *PvNF-YA1* transcripts accumulate at higher levels in nodules of 14 days post-inoculation than in roots or young nodules of 7 dpi (Ripodas et al., 2015). Here, we used a promoter*:*reporter fusion to investigate the activity of the *PvNF-YA1* promoter in different tissues of roots and nodules. A construct comprising approximately 2 kb upstream of the translational initiation codon of *PvNF-YA1* fused to the open reading frame of *GUS* reporter gene was introduced into *P. vulgaris* roots by *A. rhizogenes*-mediated transformation. Histological staining of non-inoculated *ProPvNF-YA1*:*GUS* hairy roots revealed GUS activity in the vascular tissue of primary roots, but not in lateral root primordia (Figure 2A). GUS staining was also observed in lateral root meristem (Figure 2B), as well as in the vascular tissue of non-inoculated lateral roots (Figure 2C,E). Upon inoculation with a *R. etli* strain SC15 (carrying the *nodC*-α allele), a strong and a more intense GUS staining was detected on curled root hairs and epidermal cells surrounding the infection foci of the rhizobia susceptible zone, which contains elongating root hairs, but not in fully elongated root hairs (Figure 2D,F). At later time points, GUS staining was detected in the dividing cells of nodule primordia (Figure 2G) as well as in the central tissue of nodules formed by a *R. etli* strain carrying the *nodC*-α allele (Figure 2H).

Taken all together, the results presented in this section indicate that PvNF-YA1 is located in the nuclei of vascular tissues of primary and lateral roots, but also of rapidly dividing tissues such as lateral root meristems or nodule primordia. Importantly, *PvNF-YA1* is responsive to rhizobia, being active at early stages of the RNS (root hair curling), as well as at later stages in N-fixing nodules.

### *PvNF-YA1 and PvNF-YA9* Are Symbiotic Subunits Required for Nodule Formation and Bacterial Infection

**FIGURE 3:**
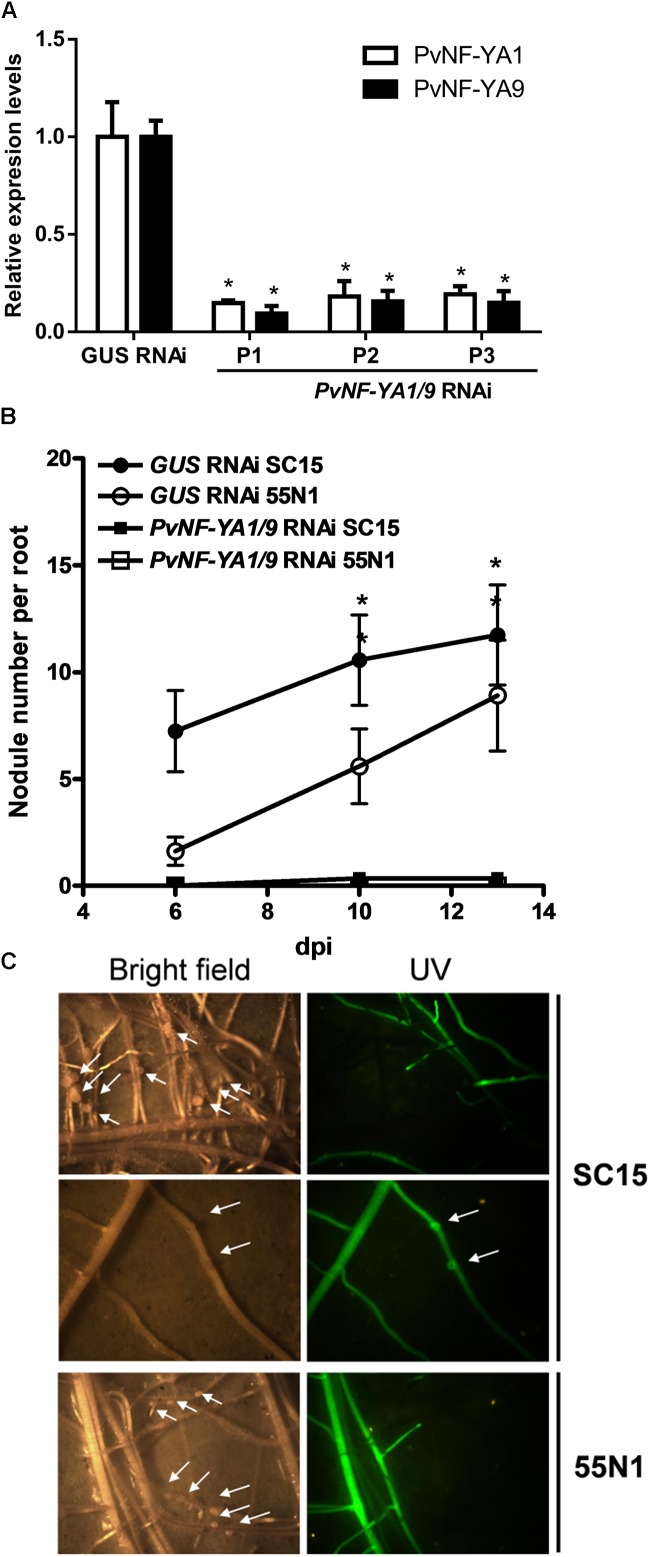
Knock-down of *PvNF-YA1/9* abolished nodule formation in *P. vulgaris*. **(A)** RT-qPCR analysis of the mRNA levels of *PvNF-YA1* (white bars) and *PvNF-YA9* (black bars) in *GUS* RNAi transgenic roots (control) and root systems collected from three independent *PvNF-YA1/9* RNAi plants (P1, P2, and P3). Expression data were normalized to *PveEF1α* gene and are presented relative to the control roots. The error bars represent the SD of three technical replicates. Asterisks indicate statistical significant differences in a *t*-test with *p* < 0.05 comparing the values of each gene in each *PvNF-YA1/9* RNAi plant with the control *GUS* RNAi. **(B)** Number of nodules formed per root in *GUS* RNAi (control) and *PvNF-YA1/9* RNAi composite plants inoculated with the strains of *R. etli* SC15 and 55N1. Non-transgenic roots were removed before inoculation, thus only nodules formed in transgenic fluorescent hairy roots were registered. The error bars represent the SEM. Data are the average of three independent biological replicates with more than 60 transgenic roots for each condition. Asterisks indicate statistical significant differences in a *t*-test with *p* < 0.05 comparing. **(C)** Images illustrating the absence of nodules in fluorescent roots of plants transformed with the *PvNF-YA1/9* RNAi construct and their presence in the non-fluorescent roots that were not excised from the root system. The arrows mark the presence of two nodule primordia or bumps. Pictures were taken at 10 dpi with strains SC15 (upper panels) or 55N1 (lower panels).

We have previously shown that both *PvNF-YA1* and *PvNF-YA9* mRNA levels increased in roots at early stages of the symbiotic interaction (i.e., 24 hpi) with *R. etli*. However, *PvNF-YA1* transcript levels increased specifically upon inoculation with the more efficient *nodC*-α strain SC15, whereas *PvNF-YA9* mRNAs accumulated at higher levels in response to both *nodC*-α and *nocC*-δ strains as compared with non-inoculated roots (Ripodas et al., 2015). Additionally, Laloum et al. (2014) showed that the orthologs of *PvNF-YA1* and *PvNF-YA9* in *M. truncatula*, *MtNF-YA2* and *MtNF-YA1*, respectively, have partially redundant functions during RNS (Laloum et al., 2014). Thus, we questioned whether these two symbiotic NF-YA members play functions during the *P. vulgaris* ×*R. etli* interaction. Anticipating that PvNF-YA1 and PvNF-YA9 might display redundant functions, an RNAi construct based on a sequence of the 3^′^ untranslated region (UTR) of *PvNF-YA1* mRNA that is highly similar to the 3^′^UTR of the PvNF-YA9 mRNA (Supplementary Figure S1) was designed to obtain *P. vulgaris* hairy roots with simultaneous reduction in *PvNF-YA1* and *PvNF-YA9* transcript levels. Transgenic roots were distinguished by visualization of the fluorescence emitted by the GFP protein, which is expressed under the control of the *rolD* promoter present in the T-DNA of the vector used for RNAi (see section “Materials and Methods”). Non-fluorescent roots were removed before collection of the tissue. RT-qPCR experiments verified that hairy roots from three independent *PvNF-YA1/9* RNAi plants exhibit reduced PvNF-YA1 and PvNF-YA9 mRNA levels (∼80%), as compared with control roots transformed with a *GUS* RNAi construct (Figure 3A). The fragment used for RNAi includes the *PvNF-YA1* binding sites for the microRNA miR169, a post-transcriptional mechanism that regulates most NF-YA family members (Combier et al., 2006; Ripodas et al., 2015). Notably, expression of this RNAi construct did not affect the expression of other members of NF-YA family (Supplementary Figure S2). Specific members of *L. japonicus* and Arabidopsis NF-YA families (e.g., *LjNF-YA1*, *AtNF-YA2*, and *AtNF-YA10*) have been implicated in root development (Soyano et al., 2013; Sorin et al., 2014), thus we performed a phenotypic analysis of the root system. Knock-down of *PvNF-YA1/A9* RNAi did not alter the length of primary and lateral roots or the density of lateral roots (Table 1). Under symbiotic conditions, GFP-expressing transgenic *PvNF-YA1/9* RNAi roots barely developed nodules either after inoculation with *nodC-α* (SC15) or *nodC-*δ (55N1) strains (Figure 3B,C). Only two bumps were detected in over 100 *PvNF-YA1/9* RNAi roots examined in three independent experiments (Figure 2C). On the other hand, *GUS* RNAi roots or non-transgenic roots (non-fluorescent roots) that were not removed from the roots systems of *PvNF-YA1/9* RNAi composite plants, developed a higher number of nodules when inoculated with the more efficient strain of *R. etli* SC15 as compared with the less efficient strain 55N1 (Figure 3B,C first and third panels), as previously described (Blanco et al., 2009; Zanetti et al., 2010). These results indicate that *PvNF-YA1* and *PvNF-YA9* play key functions in nodule formation.

**Table 1:** Phenotypic analysis of root architecture in *GUS* RNAi and *PvNF-YA1/9* RNAi roots.

	Primary root	Lateral root	Lateral root
	length^a^ (cm)	length^b^ (cm)	density^c^ (n/cm )
*GUS* RNAi	9.95 ± 0.40	2.20 ± 0.20	3.0 ± 0.20
*PvNF-YA1/9* RNAi	9.15 ± 0.87	2.10 ± 0.15	2.46 ± 0.22


*^a^Number of primary roots analyzed > 50. ^b^Number of lateral roots analyzed > 100. ^c^Density is expressed as the number of lateral roots per cm of primary root. Number of primary roots analyzed > 30. Values between GUS RNAi and PvNF-YA1/9 RNAi roots were not significantly different in an unpaired t-test with P < 0.05.*

**FIGURE 4:**
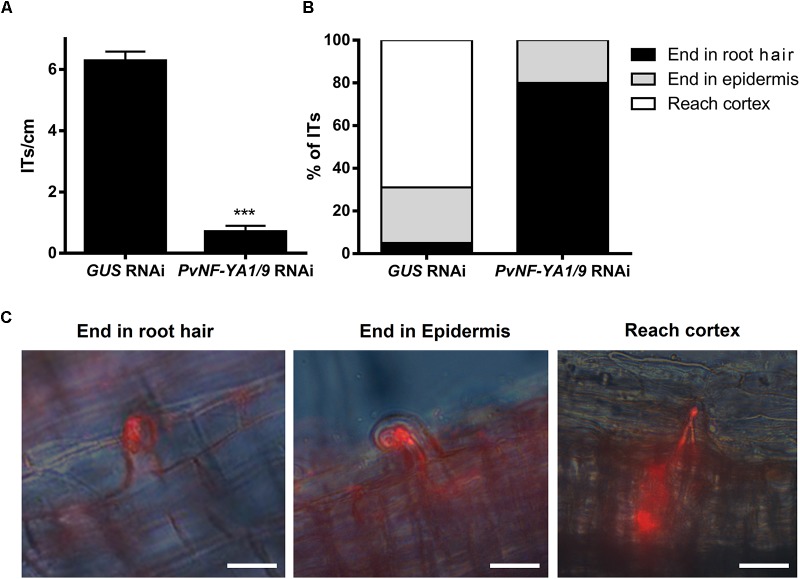
Knock-down of *PvNF-YA1/A9* impaired the initiation and progression of infection events. **(A)** Number of ITs formed per centimeter of root in *GUS* RNAi and *PvNF-YA1/9* RNAi composite plants. ITs were quantified at 5 dpi with a *R. etli* strain CFNx5 (*nodC-*α) expressing the DsRed protein. Root segments from the susceptible zone were excised and visualized under fluorescent microscope. The number of roots segments analyzed was at least 50. Asterisks denote statistical significant differences in a *t*-test with *p* < 0.001. Data are the average of three independent biological replicates. **(B)** ITs formed were classified as events that reached the cortex (white bars), the epidermis (gray bars) or aborted in the root hair (black bars) and expressed as a percentage of the total of infection events. The number of ITs that abort in the root hairs or in the epidermis were significantly higher in the *PvNF-YA1/9* RNAi plants than in the *GUS* RNAi in a *t*-test with *p* < 0.05, whereas no events were recorded that reach the cortex in *PvNF-YA1/9* RNAi plants. Data are the average of three independent biological replicates. More than 50 root segments were analyzed in each biological replicate. **(C)** Images illustrating the classification shown in **B** as ITs that aborted in the root hair, in the epidermal cells, or that reached the cortical cells of *GUS* RNAi roots.

The availability of rhizobia strains expressing fluorescent markers allowed us to evaluate the number of infection events formed, as well as their progression toward cortical cells by fluorescence microscopy. Simultaneous knock-down of *PvNF-YA1/A9* in *P. vulgaris* roots provoked an important reduction in the density of ITs as compared with control roots transformed with the *GUS* RNAi construct (Figure 4A). In addition, whereas in control plants 70% of ITs progressed and reached the cortical cell layer, nearly 80% of ITs formed in *PvNF-YA1/9* RNAi roots aborted in the root hair, 20% of them ended at the base of the epidermal cells, but none progressed to the cortex (Figure 4B,C). These results suggested these two members of the NF-YA family of *P. vulgaris* would be required for both the initiation and progression of rhizobial infection events.

### Knock-Down of *PvNF-YA1/A9* Impairs Induction of Early Nodulins and Cell Cycle Genes

**FIGURE 5:**
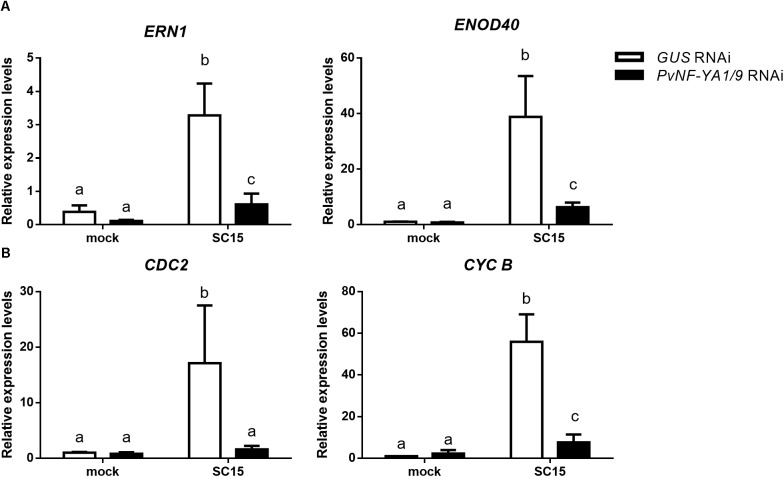
Rhizobial induction of early nodulins and cell cycle genes is compromised by silencing of *PvNF-YA1*/*A9*. Roots expressing *GUS* (white bars) or *PvNF-YA1/9* RNAi (black bars) constructs were inoculated with the *R. etli* strain SC15 or mock inoculated with the media used to growth the rhizobia (mock). At 24 hpi, transcript levels of **(A)** nodulin and **(B)** cell cycle genes were determined by RT-qPCR. Values were normalized to *PvEF1α* and presented relative to the values of the mock-inoculated *GUS* RNAi roots. Error bars represent the SEM and different letters indicate that values are significantly different in a *t*-test with *p* < 0.05. Data are the average of three independent biological replicates, each performed with at least five independent plants.

Considering the symbiotic phenotype observed in *PvNF-YA1/9* RNAi roots and the well-known function of NF-Y heterotrimers as transcriptional modulators, we investigated whether knock-down of *PvNF-YA1/9* affected the rhizobial-induced accumulation of nodulation marker transcripts. We selected *ERN1* (*ERF Required for Nodulation 1*), a mRNA encoding a transcription factor of the ERF (Ethylene Response Factor) family required for nodulation and rhizobial infection (Middleton et al., 2007) and *ENOD40* (*Early Nodulin 40*), a highly structured RNA required for cortical cell divisions that will form nodule primordia (Crespi et al., 1994). Previously, we have shown that these transcripts accumulated in *P. vulgaris* roots upon infection with *R. etli* strain SC15 and 55N1 (Zanetti et al., 2010; Mazziotta et al., 2013). RNA-sequencing data confirmed that these two nodulation marker transcripts accumulated to higher levels in wild type roots upon inoculation with either strain SC15 or 55N1 (Supplementary Figure S3; Dalla Via et al., 2015). Consistently with this observation, RT-qPCR experiments conducted in this study showed that both *ERN1* and *ENOD40* transcript levels increased more than 20 folds in control (*GUS* RNAi) hairy roots at 24 hpi as compared with non-inoculated *GUS* RNAi roots. However, *PvNF-YA1/9* RNAi roots failed to increase *ERN1* transcripts, and showed only a moderate increase (five folds) in *ENOD40* transcript levels in response to rhizobial infection (Figure 5A and Supplementary Figure S4). These results suggest that *PvNF-YA1/9* subunits might regulate directly or indirectly the expression of early nodulation genes in response to rhizobia.

In the context of nodulation, it has been shown that genes involved in cell cycle progression are activated at early time points after inoculation with rhizobia (Soyano et al., 2013; Breakspear et al., 2014). Transcripts of two cell cycle genes, *cyclin B* (*CYCB*) and *CDC2*, accumulated to higher levels in *P. vulgaris* roots in response to strain SC15, but not in response to strain 55N1 (Zanetti et al., 2010; Supplementary Figure S3). Moreover, we have shown that *PvNF-YC1* is required for accumulation of *CYCB* and *CDC2* mRNAs during the symbiosis of *P. vulgaris* with *R. etli* (Zanetti et al., 2010). Thus, we assessed whether silencing of *PvNF-YA1/A9* affected rhizobial-induced transcript accumulation of these cell cycle genes. As shown in Figure 5B, *PvNF-YA1/9* RNAi roots failed to accumulated higher transcript levels of *CDC2* and *CYCB* upon rhizobial infection in contrast with that observed in *GUS* RNAi roots. These results were consistently observed in three independent biological replicates (Supplementary Figure S4), suggesting a possible role of these NF-YA members in the re-activation of mitotic divisions of those cells committed for symbiosis, which is required for initiation of nodule organogenesis.

### Overexpression of *PvNF-YA1*, but Not *PvNF-YA9*, Enhances the Symbiotic Outcome of a Less Efficient and Competitive Rhizobial Strain

**FIGURE 6:**
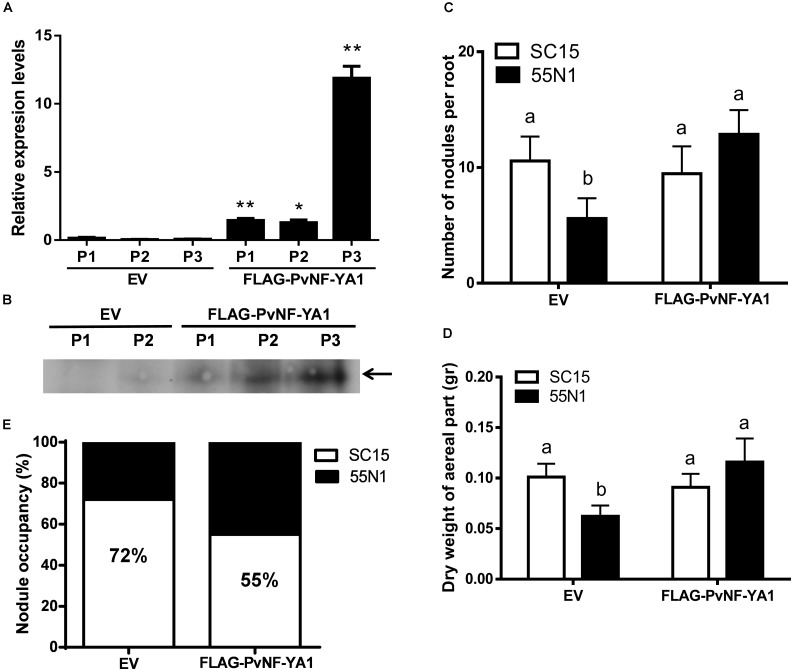
Overexpression of PvNF-YA1 enhances nodulation of the less efficient *R. etli* strain 55N1 and alters nodule occupancy in *P. vulgaris.* **(A)** *PvNF-YA1* mRNA levels measured by RT-qPCR in control plants [empty vector (EV)] or independent roots of FLAG-PvNF-YA1. Error bars represent the SD of two technical replicates. Expression data were normalized to *PveEF1α* and presented as relative to EV. P1, P2, and P3 indicate the plant number. Asterisks indicate statistical significant differences in a *t*-test with *P* < 0.05. **(B)** Western blot analysis with α-FLAG antibody on root extracts from EV and FLAG-PvNF-YA1 plants. The band observed in the last three lines corresponds to the FLAG-PvNF-YA1 fusion of 38 kDa (arrow). P1, P2, and P3 correspond to the pool of transgenic roots of three different plants. **(C)** Number of nodules per root developed in control roots (EV) or FLAG-PvNF-YA1 at 10 dpi with strains of *R. etli* SC15 (white bars) or 55N1 (black bars). The error bars represent the SEM of the number of nodules formed per root present in more than 50 independent transgenic roots. Different letters indicate significantly different values in a *t*-test with *p* < 0.05. Data are the average of three independent biological experiments. **(D)** Dry weight of the aerial tissue of EV or FLAG-PvNF-YA1 composite plants at 21 dpi with strains of *R. etli* SC15 or 55N1. Error bars represent the SEM of at least 20 independent plants. Different letters indicate significantly different values in a *t*-test with *p* < 0.05. Data are the average of three independent biological experiments. **(E)** Percentage of nodule occupancy by strains SC15 (white) and 55N1 (black) in co-inoculation experiments. Nodule occupancy was determined at 21 dpi by examination of *nodC* polymorphic profiles of bacteria isolated from more than 100 individual nodules collected from a minimum of 10 co-inoculated plants for each construction. The results are the average of three independent biological experiments.

The function of *PvNF-YA1* and *PvNF-YA9* in the establishment of the interaction between *P. vulgaris* and different rhizobia strains was investigated using transgenic roots that express a FLAG-tagged version of these subunits under the control of the nearly constitutive promoter Cauliflower Mosaic Virus 35S (CaMV35S), designated as FLAG-PvNF-YA1 and FLAG-PvNF-YA9. Roots transformed with the FLAG-PvNF-YA1 construct exhibited 2–10 fold times higher levels of *PvNF-YA1* mRNAs than control roots transformed with the empty vector (EV) as revealed by RT-qPCR in three different composite plant (Figure 6A). Western blot experiments using anti-FLAG antibodies verified the accumulation of the FLAG-PvNF-YA1 proteins and showed a relatively good correlation with *PvNF-YA1* transcript levels in the three independent composite plants analyzed here (Figure 6B). Phenotypic analysis of FLAG-NF-YA1 composite plants reveals that overexpression of *PvNF-YA1* had no effect on the length of the main and lateral roots (Table 2). FLAG-PvNF-YA1 roots showed a slight increase in the number of lateral roots developed by cm of root (density of lateral roots) compared to control plants transformed with the EV; however, this difference was not statistically significant (Table 2).

**Table 2:** Phenotypic analysis of root architecture in EV and FLAG-PvNF-YA1 roots.

	Primary root	Lateral root	Lateral root
	length^a^ (cm)	length^b^ (cm)	density^c^ (n/cm)
EV	11.21 ± 0.45	1.18 ± 0.09	4.7 ± 0.5
FLAG-PvNF-YA1	10.15 ± 0.71	1.24 ± 0.12	5.8 ± 0.8


*^a^Number of primary roots analyzed > 50. ^b^Number of lateral roots analyzed > 50. ^c^Density is expressed as the number of lateral roots per cm of primary root. Number of primary roots analyzed > 25. Values between EV and FLAG-PvNF-YA1 roots were not significantly different in an unpaired t-test with P < 0.05.*

To evaluate whether overexpression of *PvNF-YA1* affects the symbiotic outcome of *P. vulgaris* with different *R. etli* strains, FLAG-PvNF-YA1 and EV roots were inoculated with either the *nodC*-α strain SC15 or the *nodC*-δ strain 55N1 of *R. etli.* Overexpression of NF-YA1 did not produce a significant difference in the number of nodules per root formed by strain SC15 or in the dry weight of the aerial part (Figure 6C,D, white bars). However, upon inoculation with strain 55N1, FLAG-PvNF-YA1 roots formed a significantly greater number of nodules per root than control EV roots (Figure 6C, black bars). This increase in the number of nodules was correlated with an increase in the dry weight of the aerial part of the plant, which could be associated with increased efficiency of N fixation in the FLAG-PvNF-YA1 plants inoculated with 55N1 (Figure 6D, black bars). Notoriously, both parameters—nodule number and dry weight—of FLAG-PvNF-YA1 plants inoculated with 55N1 reached values comparable to those measured when plants were challenged with SC15. All these results show the same trend previously reported in plants overexpressing PvNF-YC1 (Zanetti et al., 2010). On the other hand, constitutive expression of FLAG-PvNF-YA9 in *P. vulgaris* roots did not affect the number of nodules formed by neither SC15 nor 55N1 strains of *R. etli* (Supplementary Figures S5A,B).

Previous studies have shown that Mesoamerican accession of *P. vulgaris* are preferentially nodulated by strains of rhizobia carrying the *nodC-*α allele when co-inoculated with strains with de *nodC*-δ allele (Aguilar et al., 2004). Moreover, overexpression of the PvNF-YC1 subunit was sufficient to alter nodule occupancy by these strains (Zanetti et al., 2010). Based on these previous observations and having found that the overexpression of *PvNF-YA1* affects the interaction with the less efficient and competitive strain 55N1, we proceeded to assess the role of PvNF-YA1 and PvNF-YA9 in the strain preference observed in Mesoamerican beans. Roots of composite plants were co-inoculated with an equicellular mixture of *R. etli* strains SC15 and 55N1. Nodules were collected at 21 dpi and the identity of the strain contained within individual nodules was evaluated by analyzing the phenotype of the strains growing in Congo red-YEM (Yeast Extract Mannitol) agar plates or the genotype by detection of the polymorphism of the *nodC* gene as previously described (Zanetti et al., 2010; Supplementary Figure S6). In control roots, 72% of the nodules were occupied by strain SC15 and only 28% by strain 55N1. Interestingly, FLAG-PvNF-YA1 roots showed an increase of 17% in the nodule occupancy by the strain 55N1 as compared to the control plants (Figure 6E), indicating that constitutive and ectopic expression of PvNF-YA1 is sufficient to alter nodule occupancy in Mesoamerican beans. On the contrary, overexpression of FLAG-PvNF-YA9 did not alter nodule occupancy by SC15 and 55N1 strains (Supplementary Figure S5C). Thus, these results suggest that PvNF-YA1, but not PvNF-YA9, might play functions in the strain preference observed in Mesoamerican beans by the strains carrying the *nodC-*α allele.

### *PvNF-YB7* Is Expressed in the Nodule Central Tissue and Required for the Selection of the Highly Efficient Nodulation Strain of *R. etli*

**FIGURE 7:**
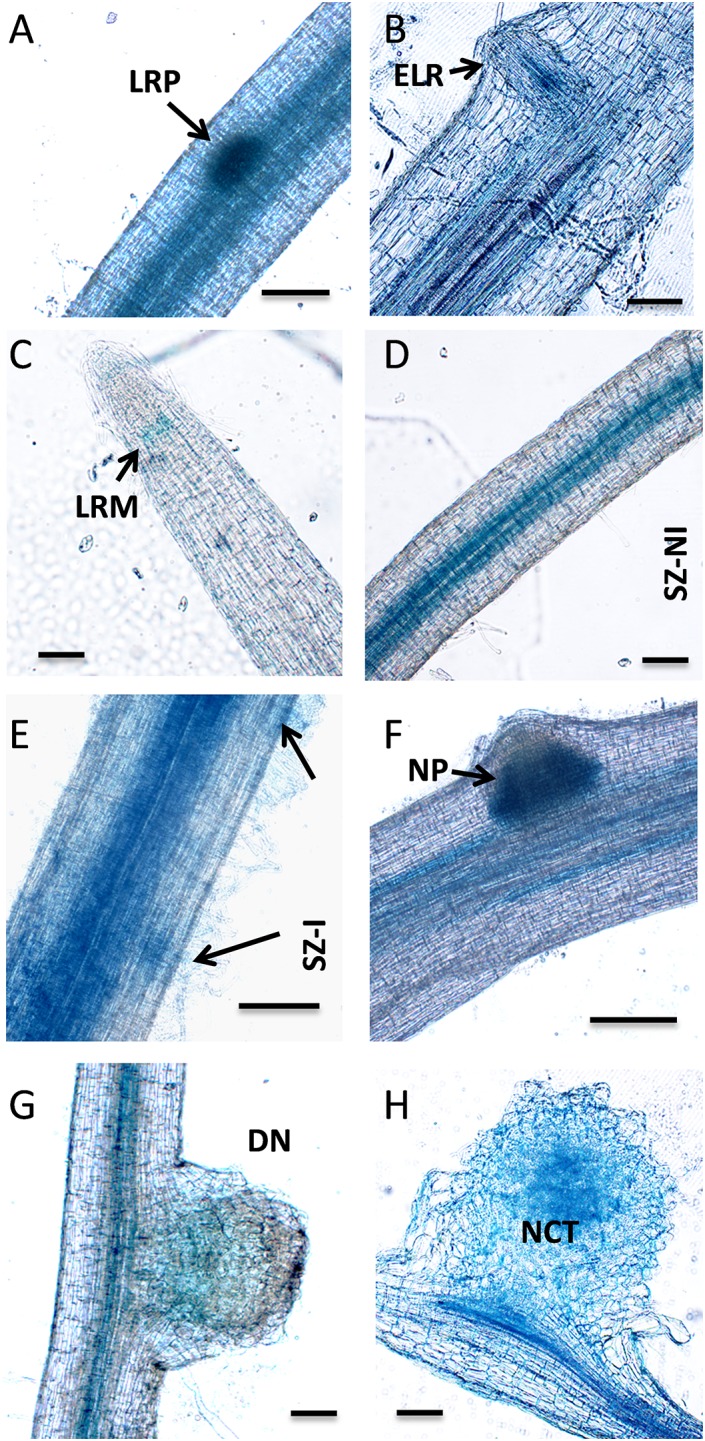
Tissue-specific expression analysis of *PvNF-YB7* in roots and nodules of *P. vulgaris.* Histochemical GUS staining of roots and nodules transformed with the *ProPvNF-YB7:GUS* construct. **(A)** Non-inoculated (NI) primary root which containing a lateral root primordia (LRP). **(B)** Thin section of a primary root containing an emerged lateral root (ELR). **(C)** Lateral root meristem (LRM) of a NI lateral root. **(D)** Susceptible zone (SZ) of a NI lateral root. **(E)** SZ of lateral root inoculated (I) with *R. etli* strain SC15. Arrows point to infection sites. **(F)** Nodule primordia (NP) of 6 dpi, **(G)** developing nodule (DN) of 14 dpi, and **(H)** mature nodule of 21 dpi formed by strain SC15. NCT: nodule central tissue. Pictures are representative of images observed in more than three independent experiments. Bars: 200 μm.

**FIGURE 8:**
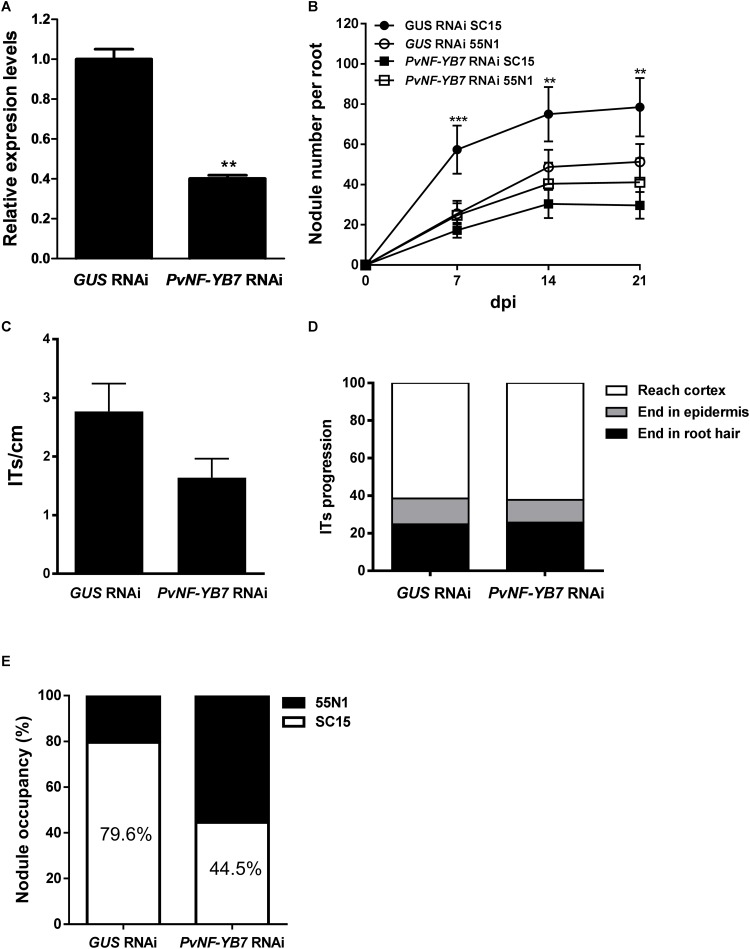
Knock-down of *PvNF-YB7* reduces nodule formation and occupancy by the high efficient *R. etli* strain SC15. **(A)** RT-qPCR analysis of the mRNA levels of *PvNF-YB7* in *GUS* RNAi transgenic roots (control) and in roots from three independent *PvNF-YB7* RNAi plants. Expression data were normalized to *PveEF1α* gene and are presented relative to the control roots. Results are the average of two or three technical replicates. The error bars represent the SD. Asterisks indicate statistical significant differences in a *t*-test with *p* < 0.01. The results are representative of three independent biological experiments. **(B)** Number of nodules per root formed in *GUS* (control) and *PvNF-YB7* RNAi composite plants inoculated with the strains of *R. etli* SC15 and 55N1. The error bars represent the SEM. Data are the average of three independent biological replicates (*n* > 50 for each condition). Two or three asterisks indicate statistical significant differences in a *t*-test with *p* < 0.01 or *p* < 0.001. **(C)** Number of ITs formed per centimeter of root in *GUS* and *PvNF-YB7* RNAi composite plants. ITs were quantified at 5 dpi with a *R. etli* strain CFNx5 (*nodC*-α) expressing the DsRed protein. Data are the average of three independent biological replicates each with more than 50 roots segments from the susceptible zone. No statistical significant differences were found in a *t*-test with *p* < 0.05. Data are the average of three independent biological replicates. **(D)** ITs were classified as events that reached the cortex (white bars), the epidermis (gray bars), or aborted in the root hair (black bars) and expressed as a percentage of the total infection events. Data are the average of three independent biological replicates each with more than 50 independent roots segments. **(E)** Percentage of nodule occupancy by strains SC15 (white) and 55N1 (black) in co-inoculation experiments in *GUS* and *PvNF-YB7* RNAi roots. Nodule occupancy was determined at 21 dpi by examination of *nodC* polymorphic profiles of bacteria isolated from more than 100 individual nodules obtained from at least 10 composite plants. Results are the average of three independent biological replicates.

As already mentioned, the PvNF-YB7 subunit forms a heterotrimer *in planta* with PvNF-YA1 and PvNF-YC1 subunits, and *PvNF-YB7* mRNAs accumulate at higher levels in symbiotic nodules upon inoculation with *R. etli*. Thus, we focused our analysis in this specific member of the PvNF-YB family. A spatial expression analysis using a promoter:GUS construct was performed in transgenic hairy roots of *P. vulgaris*. Roots transformed with the *PromPvNF-YB7:GUS* construct exhibited GUS staining in the vascular tissue of the elongation zone of primary roots, as well as in the lateral root primordia (Figure 7A). GUS staining was also detected in the vasculature of emerged lateral roots (Figure 7B). In already developed lateral roots, expression of *PvNF-YB7* was observed in a small number of cells of the lateral root meristem as well as in the vasculature of the susceptible zone (Figure 7C,D). Under symbiotic conditions, GUS staining was observed in the curled root hairs of the susceptible zone (Figure 7E), in the dividing tissue of nodule primordia (Figure 7F), as well as in the base of developing nodule (Figure 7G) and in the central tissue of nodules developed by the *R. etli* *nodC*-α strain SC15 (Figure 7H). These results indicate that the *PvNF-YB7* promoter is active during nodule formation and support the notion that this subunit plays a relevant function in the establishment of symbiotic nodules. Then, we investigated the functional relevance of *PvNF-YB7* in the establishment of an efficient symbiotic interaction and in the strain preference in Mesomearican beans using RNAi-mediated gene silencing. Expression of an RNAi construct specifically designed to silence *PvNF-YB7* in *P. vulgaris* roots resulted in a significant reduction (>40%) of *PvNF-YB7* mRNA levels (Figure 8A). Interestingly, *PvNF-YB7* RNAi roots inoculated with the *nodC*-α strain SC15 developed nearly half of the nodules formed in control *GUS* RNAi roots; however, when inoculated with strain 55N1, *PvNF-YB7* RNAi and *GUS* RNAi roots developed a similar number of nodules (Figure 8B), indicating that PvNF-YB7 subunit might be one of the molecular components of *P. vulgaris* that are required for the establishment of an interaction with high efficient strains of *R. etli*. Rhizobial infection was slightly, but not significantly reduced by knock-down of *PvNF-YB7* as determined by the frequency of ITs formed by an RFP labeled *R. etli* strain carrying the *nodC*-α alelle (Figure 8C). In addition, progression of the infection events was not altered by the reduction in *PvNF-YB7* levels (Figure 8D), suggesting that this member of the NF-YB family is not strictly required for the initiation and elongation of ITs. Since silencing of *PvNF-YB7* affected nodule formation with SC15 but not with 55N1, we questioned whether this silencing could affect the strain selectivity observed in Mesoamerican beans. Notably, upon co-inoculation with a mixture of strains SC15 and 55N1, the occupancy of the nodules by strain SC15 was reduced in more than 35% in *PvNF-YB7* RNAi roots as compared with *GUS* RNAi roots (Figure 8E). All together, the results presented here support a role for *PvNF-YB7* in nodule organogenesis triggered specifically by the high efficient strain SC15.

## Discussion

Nuclear Factor Y TFs act as heterotrimers to activate or repress expression of their target genes. Since the individual subunits of the complex are encoded by relatively large gene families in plant genomes, it is crucial to elucidate the composition of the heterotrimers that would be acting in specific tissues or during the activation of morphogenetic programs, such as rhizobial infection and nodule organogenesis, which will largely rely on the tissue-specific expression pattern of individual members of these gene families. Our previous studies have shown that *PvNF-YA1*, *PvNF-YA9*, *PvNF-YB7* and *PvNF-YC1* are expressed in nodules (Ripodas et al., 2015). Here, the use of promoter:GFP-GUS constructs revealed that the promoters of both *PvNF-YA1* and *Pv-NF-YB7* are active in infected root hairs, as well as in the central tissue of N-fixing nodules developed by the strain of *R. etli* carrying the *nodC*-*α* allele. The expression pattern of these two subunits is reminiscent of that described for *LjNF-YA1* and *LjNF-YB1* in the legume *L. japonicus*, in which *LjNF-YA1* plays a crucial role in nodule formation (Soyano et al., 2013). In addition, it is consistent with that reported for MtNF-YA1 promoter at early stages of symbiosis between *M. truncatula* and *Sinorhizobium meliloti*, in which expression was detected in infected root hairs of the susceptible zone and in the central region of developing nodules (Laporte et al., 2014). The expression pattern of *PvNF-YA1* and *PvNF-YB7* partially overlap in roots under non-symbiotic conditions. Expression of *PvNF-YB7*, but not PvNF-YA1, was detected in lateral root primordia, suggesting that they might be acting in different heterotrimeric NF-Y complex at initial steps of lateral root formation, prior emergence. In already emerged lateral roots, expression of *PvNF-YA1* and *PvNF-YB7* overlap in vascular and meristematic tissue, suggesting that both subunits might be part of a heterotrimer involved in post-emergence lateral root growth. This speculation is consistent with the role assigned for LjNF-YA1 and LjNF-YB1 subunits in lateral root growth (Soyano et al., 2013). Since PvNF-YA1 and PvNF-YB7, together with PvNF-YC1, have been shown to be part of the same hetertorimeric complex *in planta* and their expression overlap in developing nodules formed by the more efficient strain of *R. etli*, it is possible to speculate that these two subunits might act in concert to promote nodule formation and/or development during the high efficient interaction established between Mesoamerican beans and strains of *R. etli* carrying the *nodC*-*α* allele. This speculation is supported by the phenotype observed in plants with altered levels of these NF-Y subunits. The results presented here revealed that both *PvNF-YA1/A*9 and *PvNF-YB7* RNAi roots showed a significant reduction in the number of nodules formed by the strain SC15. A previous study indicated that silencing of *PvNF-YC1* also prevented nodule formation by this *R. etli* strain (Zanetti et al., 2010), indicating that the three subunits might act in concert in nodule formation and development triggered by the more efficient strain SC15. On the other hand, when plants were inoculated with the less efficient strain 55N1, nodule formation was also diminished in *PvNF-YA1/9* RNAi and *PvNF-YC1* RNAi roots, but not in *PvNF-YB7* RNAi roots. These results indicate that other NF-YB subunits, e.g., PvNF-YB10 or PvNF-YB12, which are also expressed in nodules (Ripodas et al., 2015), might fulfill this function when plants are challenged by a strain that is less efficient in nodule formation, presumably by heterotrimerization with PvNF-YC1 and PvNF-YA1 or PvNF-YA9. This was not unexpected since expression of a RNAi designed to specifically silence *MtNF-YB16* or a less specific RNAi that simultaneous knock-down four NF-YB gene family members (*MtNF-YB16/B18/B6/B11*) in *M. truncatula* did not revealed any noticeable symbiotic phenotype upon inoculation with *S. meliloti* (Baudin et al., 2015). In addition, two independent studies based on yeast-two hybrid assays performed with Arabidopsis NF-Y subunits revealed that NF-YB subunits tend to be more promiscuous in heterotrimer formation than NF-YA and NF-YC subunits (Calvenzani et al., 2012; Hackenberg et al., 2012). The fact that *PvNF-YB7* RNAi plants form nodules with both *R. etli* strains points toward a low degree of specificity in the NF-YB association for nodule formation, as previously proposed for the *M. truncatula* ×*S. meliloti* interaction (Baudin et al., 2015). However, the observation that *PvNF-YB7* seems to be required for a high efficient nodule formation leads to speculate that the symbiotic outcome, at least in the case of *P. vulgaris* ×*R. etli* interaction, might depend on strict NF-Y heterotrimer formation.

**FIGURE 9:**
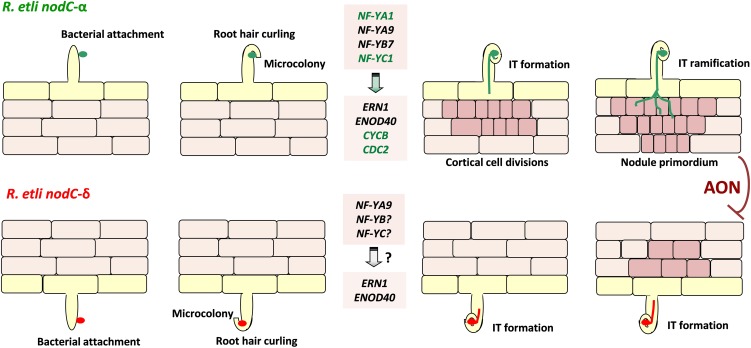
Model illustrating the proposed function of the PvNF-YA1/-YB7/-YC1 heterotrimer in the strain preference observed in the *P. vulgaris* × *R. etli* interaction. Perception of *R. etli* strains *nodC*-α (represented in green, upper panels) by Mesoamerican *P. vulgaris* plants results in the root hair curling that entrap bacteria to form a microcolony. This perception also rapidly (within 24 hpi) and locally induces expression of genes encoding the PvNF-YA1 (and PvNF-YA9) and PvNF-YC1 subunits, which form a hetrotrimeric transcription factor with PvNF-YB7. Heterotrimer formation activates the expression of nodulation (*ERN1* and *ENOD40*) and the cell cycle (*CYCB* and *CDC2*) genes, promoting infection thread (IT) formation and cortical cell divisions. The IT progresses, ramifies, and reaches the nodule primordium to produce a functional nodule occupied by the *nodC*-α strain. On the other hand, perception of *nodC*-δ strains (represented in red, lower panels) leads to root hair curling, microcolony formation, and induction of *PvNF-YA9*, *ERN1*, and *ENOD40* in a similar way that *nodC*-α strains. However, induction of *PvNF-YA1* and *PvNF-YC1*, as well as *CYCB* and *CDC2*, is not observed at 24 hpi, delaying cortical cell divisions and the progression of ITs that contain the *nodC*-δ strain. As a consequence of these local responses, when both strains are present (e.g., in coinoculation experiments or in the field when both bacteria are present in the soil with even abundance) nodules are mainly occupied by the *nodC*-α strains. Since nodulation is systemically repressed by a systemic mechanisms referred as autoregulation of nodulation (AON), new nodule formation triggered by the *nodC*-δ strain is arrested once the appropriate number of nodules is reached. In this model, genes specifically induced at 24 hpi with the *nodC*-α strain are colored in green. PvNF-YA9 and other NF-YB and NF-YC subunits, as well as ERN1 and ENOD40, are activated also by the *nodC*-δ strain.

An interesting finding of this study is the observation that overexpression of PvNF-YA1, but not of PvNF-YA9, increased the efficiency of nodule formation when plants were inoculated with the less efficient strain 55N1; moreover, it was sufficient to increase the proportion of nodules occupied by 55N1 when co-inoculated with the higher competitive strain SC15. It must also be noted that *PvNF-YA1* proved to be responsive only to strain SC15, whereas *PvNF-YA9* expression is activated by both strains (Ripodas et al., 2015). Thus, PvNF-YA9 appears to be part of the more general pathway triggered by high or low efficient strains, which might explain why overexpression of this subunit did not altered nodule number or their occupancy. On the other hand, PvNF-YA1 seems to be part of the signaling pathway activated only by the high efficient strain SC15. The phenotype observed in PvNF-YA1 overexpressing roots is reminiscent of that previously described in plants overexpressing PvNF-YC1 (Zanetti et al., 2010). Considering the fact that PvNF-YA1 and PvNF-YC1 are able to interact in the same NF-Y trimeric complex (Baudin et al., 2015), we suggest that increased levels of both PvNF-YA1 and PvNF-YC1 could help to improve the symbiotic capacity of less efficient rhizobia. Moreover, these two NF-Y subunits are part of a symbiotic heterotrimeric TF that might work in the activation of the genetic program that allows Mesoamerican plants to discriminate between strains that are more or less efficient in nodule formation. A striking result regarding nodule occupancy by SC15 and 55N1 strains was obtained in *PvNF-YB7* RNAi plants. In these plants, nodules occupied by SC15 decreased by about 35% compared to controls. This might be most likely a consequence of the reduced capacity of these plants to form nodules upon inoculation with strain SC15. Thus, we concluded that *PvNF-YB7* is required for high efficient nodule organogenesis in Mesoamerican *P. vulgaris* roots. This observation also reinforces the hypothesis that this specific NF-YB subunit might be an additional component of the genetic programs that determine the selectivity displayed by *P. vulgaris* plants for those strains that perform better in nodule formation. Based on this and previous studies (Zanetti et al., 2010; Ripodas et al., 2015), we propose a model that attempt to explain how PvNF-YA1, PVNF-YB7, and PvNF-YC1 function to control the strain preference observed in Mesoamerican accessions of *P. vulgaris* (Figure 9). In this model, specific activation of PvNF-YA1 and PvNF-YC1 in response to *nodC*-α strains, such as SC15, lead to a rapid activation of the morphological responses, such as cortical cell divisions (possibly due the specific and rapid activation of cell cycle genes) and progression of IT toward nodule primordia. These responses result in the formation of nodules occupied by *nodC*-α strains. On the other hand, *nodC*-δ strains fail to rapidly induce *PvNF-YA1* and *PvNF-YC1*, thus these morphological responses occurred slower in the presence of strains such as 55N1; as a consequence, nodules formed by these strains will be delayed. During symbiosis, existing N-fixing nodules inhibit the formation of new nodules by a mechanism referred to as autoregulation of nodulation (AON), which involves long distance signaling from root to shoot and back again (Ferguson et al., 2014). Within this context, the earliest formation of nodules occupied by a *nodC*-α strain will inhibit the formation of new nodules that might be colonized by a *nodC*-δ strain, explaining why in co-inoculation experiments or in the field, the majority of the nodules formed in Measomerican beans are occupied by *nodC*-α strains.

Infection events and their progression to the cortical cells were drastically reduced by introduction of the *PvNF-YA1/9* RNAi construct in *P. vulgaris* roots. Moreover, no ITs reaching the cortex were detected in *PvNF-YA1/9* RNAi roots, at least at the time points analyzed here, indicating that they might have aborted either in the root hair or the epidermis. Thus, these two NF-YA subunits play not only an important role in nodule organogenesis and development, but also in the epidermal and/or cortical responses that lead to a successful infection of the nodule. This phenotype resembles that previously reported by Laloum et al. (2014) in *M. truncatula* plants with reduced levels of *MtNF-YA1* and *MtNF-YA2* (the orthologs of *PvNF-YA9* and *PvNF-YA1*, respectively), where both frequency and progression of ITs were impaired. This phenotype is more pronounced than that observed in *M. truncatula* that are null mutants for the *MtNF-YA1* allele, which exhibited an increased number of ITs, but the morphology of ITs was abnormal and IT growth was arrested (Laporte et al., 2014). In contrast, *L. japonicus* plants with reduced levels of a single NF-YA subunit, LjNF-YA1 (the ortholog of PvNF-YA9 and MtNF-YA1 of *P. vulgaris* and *M. truncatula*, respectively), produced a normal amount of ITs without evidenced of IT arresting (Soyano et al., 2013), suggesting some functional redundancy with other NF-YA subunits in this legume species. Based on our results and those described by others, it is possible to conclude that two symbiotic NF-YA subunits play partially redundant and essential functions in the sophisticated intracellular infection by rhizobia observed in many legume species. On the other hand, expression of the *PvNF-YB7* RNAi construct did not produce any strong phenotype in the infection by rhizobia; except by a mild, but not significant reduction in the frequency of ITs. This indicates that other NF-YB subunits of *P. vulgaris* might exert redundant functions in the genetic program that leads to this intracellular mechanism of infection.

Nuclear Factor Y subunits have been involved in the control of cell division activities during the development of lateral root organs, either lateral roots or symbiotic nodules (Zanetti et al., 2010; Soyano et al., 2013; Sorin et al., 2014). Here, we found that lateral root growth or density was not significantly affected by knock-down of *PvNF-YA1* and *PvNF-YA9*, indicating that these subunits do not seem to be required or play redundant functions with other NF-YA members during the activation of cell divisions related to the initiation or growth of lateral roots. However, rhizobial induction of cell cycle genes such as *CDC2* and *CYCB* was almost completely abolished in *PvNF-YA1/9* RNAi roots, which is well correlated with the absence of nodule formation observed in these roots. In addition, induction of *ENOD40*, which is also required for cortical cell division activity during formation of nodule primordia, is reduced in *PvNF-YA1/9* RNAi roots. These results indicate that PvNF-YA1 and PvNF-YA9 participate not only in the transcriptional activation of early markers of infection such as *ERN1*, a direct target of the symbiotic NF-Y complex (Laloum et al., 2014; Baudin et al., 2015), but also in the control of cell division activities during nodule formation. A previous study revealed that the PvNF-YC1 subunit is also involved in the control of *CDC2* and *CYCB* during nodule formation (Zanetti et al., 2010). Future experiments will help to elucidate whether these and other cell cycle related genes are direct transcriptional targets of the symbiotic complex formed by the PvNF-YA1/9, PvNF-YB7 and PvNF-YC1 in *P. vulgaris* during the interaction with high or low efficient strains of *R. etli.*

## Author Contributions

MZ, FB, and CR conceived the research and designed the experiments. CR, MC, and JC performed the experiments with help from JV. MZ, CR, and JC analyzed the experimental results. CR and MZ wrote the manuscript. All the authors discussed the results and approved the final manuscript.

## Conflict of Interest Statement

The authors declare that the research was conducted in the absence of any commercial or financial relationships that could be construed as a potential conflict of interest.

## Footnotes

1 https://www.ensa.ac.uk/
