# Supplementary material for: The PvNF-YA1 and PvNF-YB7 Subunits of the Heterotrimeric NF-Y Transcription Factor Influence Strain Preference in the Phaseolus vulgaris–Rhizobium etli Symbiosis
Source: Front Plant Sci. 2019 Feb 28;10:221. doi: 10.3389/fpls.2019.00221 (PMC6403126; doi:10.3389/fpls.2019.00221)
Supplement: Supplementary file 1 [file Table_1.DOCX]

**Table S1.** Primers used in this study.

| **Primer name** | **Sequence (5**'**-3**'**)** |
| --- | --- |
| **PvNF-YA1/A9 RNAi F** | CACCCTGTGACAAGTTTCGTTCTGTAG |
| **PvNF-YA1/A9 RNAi R** | ACACTGAATTGTCTTCTGCGTC |
| **PvNF-YA1 OE F** | CACCATGCAAACTGTTTATCTTAAAGAAC |
| **PvNF-YA1 OE R** | TCAAACTTTAAGGTTGCAGCAG |
| **promPvNF-YA1 F** | CACCGTGGTCCTT TCCTGTACTTGGTTTCTG |
| **promPvNF-YA1 R** | CCA TTAGATGATGAGAGGGGTCGGTA |
| **PvNF-YB7 RNAi F** | CACCGGTTTGCATTTAGGGTCCAA |
| **PvNF-YB7 RNAi R** | CATCATCATTCATGCCCTGGTGC |
| **promPvNF-YB7 F** | CACCGTATCTTACGGCACTCTTAAAAAACATCAA |
| **promPvNF-YB7 R** | TGT CCA TGC TAG TTA CAA TAA ATA TCA ACA |
| **qPvNF-YA1 F** | TCATTTTGGATCTTCTGTGCACAC |
| **qPvNF-YA1 R** | GCTTCTTCATCTGGTCTCATAAAGG |
| **qPvNF-YA2 F** | GTGTGGTAGGTACGTTAGGATG |
| **qPvNF-YA2 R** | CTTCTGACAATCTCTAATCTCTCTACAG |
| **qPvNF-YA3 F** | CATACATCTGATAAGGGTGGTGG |
| **qPvNF-YA3 R** | CCAAACAAACAGCAATAAATAGCAAC |
| **qPvNF-YA4 F** | ATTTCAGGCAGCCCGAACA |
| **qPvNF-YA4 R** | AAGGATGATTTGCCAGACCAGT |
| **qPvNF-YA5 F** | GCGTCTTGCTATTCAGTAAACCAC |
| **qPvNF-YA5R** | CCATATGATGATTAGCATAAACACAGCC |
| **qPvNF-YA6 F** | GGCTTTCAAATAGCACTTCCCTC |
| **qPvNF-YA6 R** | ATAGAGTACACCATGCCACTGC |
| **qPvNF-YA7 F** | TCAGGTGAAAGAGTGGAGGAAG |
| **qPvNF-YA7 R** | CCACAAACACAAGAACAAAGCCA |
| **qPvNF-YA8 F** | GGAAACCAACACCGTCTATCAG |
| **qPvNF-YA8 R** | GGATGACTTCCCCATGTAACAC |
| **qPvNF-YA9 F** | AGGTTTCGATGGCTTTTCTGT |
| **qPvNF-YA9 R** | CATCTAGTGGGCATGGTGATAC |
| **qPvNF-YB7 F** | GATGGAAGGGGAGAAAACTGC |
| **qPvNF-YB7 R** | CCCTATGTGCATCCACAATTACC |
