# Supplementary material for: The PvNF-YA1 and PvNF-YB7 Subunits of the Heterotrimeric NF-Y Transcription Factor Influence Strain Preference in the Phaseolus vulgaris–Rhizobium etli Symbiosis
Source: Front Plant Sci. 2019 Feb 28;10:221. doi: 10.3389/fpls.2019.00221 (PMC6403126; doi:10.3389/fpls.2019.00221)
Supplement: Supplementary file 2 [file Presentation_1.PPTX]

## Slide 1
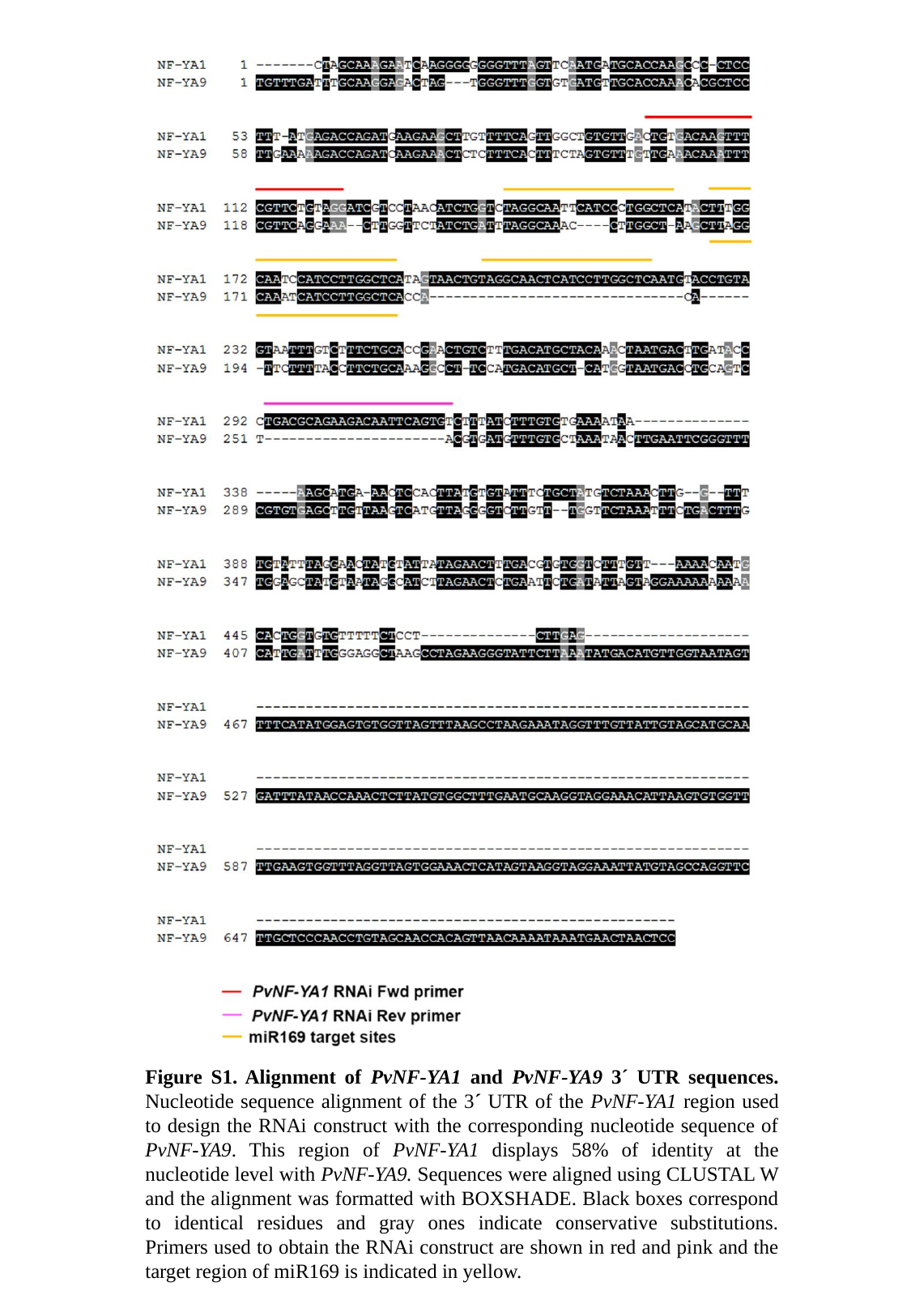

Figure S1. Alignment of PvNF-YA1 and PvNF-YA9 3´ UTR sequences. Nucleotide sequence alignment of the 3´ UTR of the PvNF-YA1 region used to design the RNAi construct with the corresponding nucleotide sequence of PvNF-YA9. This region of PvNF-YA1 displays 58% of identity at the nucleotide level with PvNF-YA9. Sequences were aligned using CLUSTAL W and the alignment was formatted with BOXSHADE. Black boxes correspond to identical residues and gray ones indicate conservative substitutions. Primers used to obtain the RNAi construct are shown in red and pink and the target region of miR169 is indicated in yellow.

## Slide 2
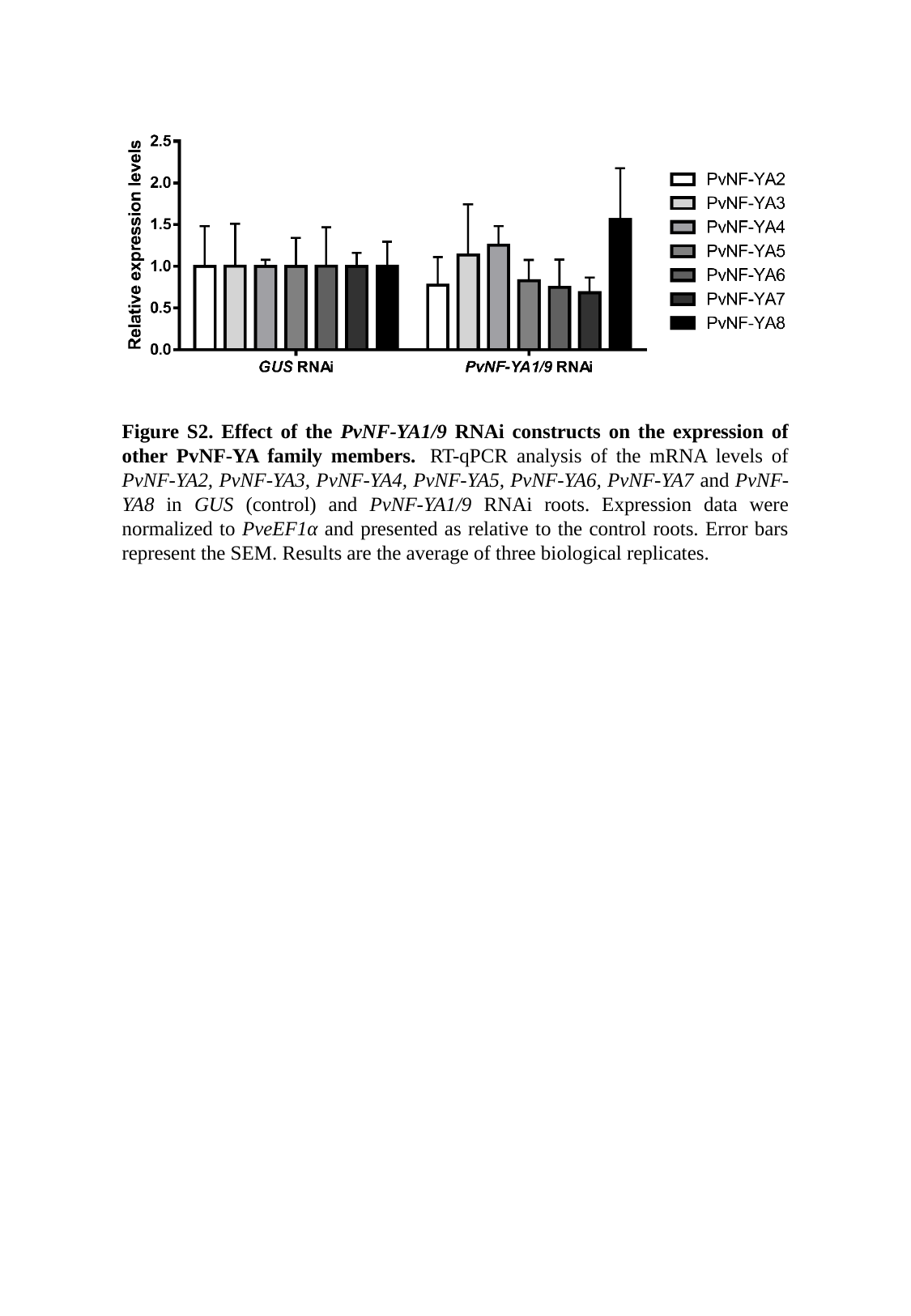

Figure S2. Effect of the PvNF-YA1/9 RNAi constructs on the expression of other PvNF-YA family members.  RT-qPCR analysis of the mRNA levels of PvNF-YA2, PvNF-YA3, PvNF-YA4, PvNF-YA5, PvNF-YA6, PvNF-YA7 and PvNF-YA8 in GUS (control) and PvNF-YA1/9 RNAi roots. Expression data were normalized to PveEF1α and presented as relative to the control roots. Error bars represent the SEM. Results are the average of three biological replicates.

## Slide 3
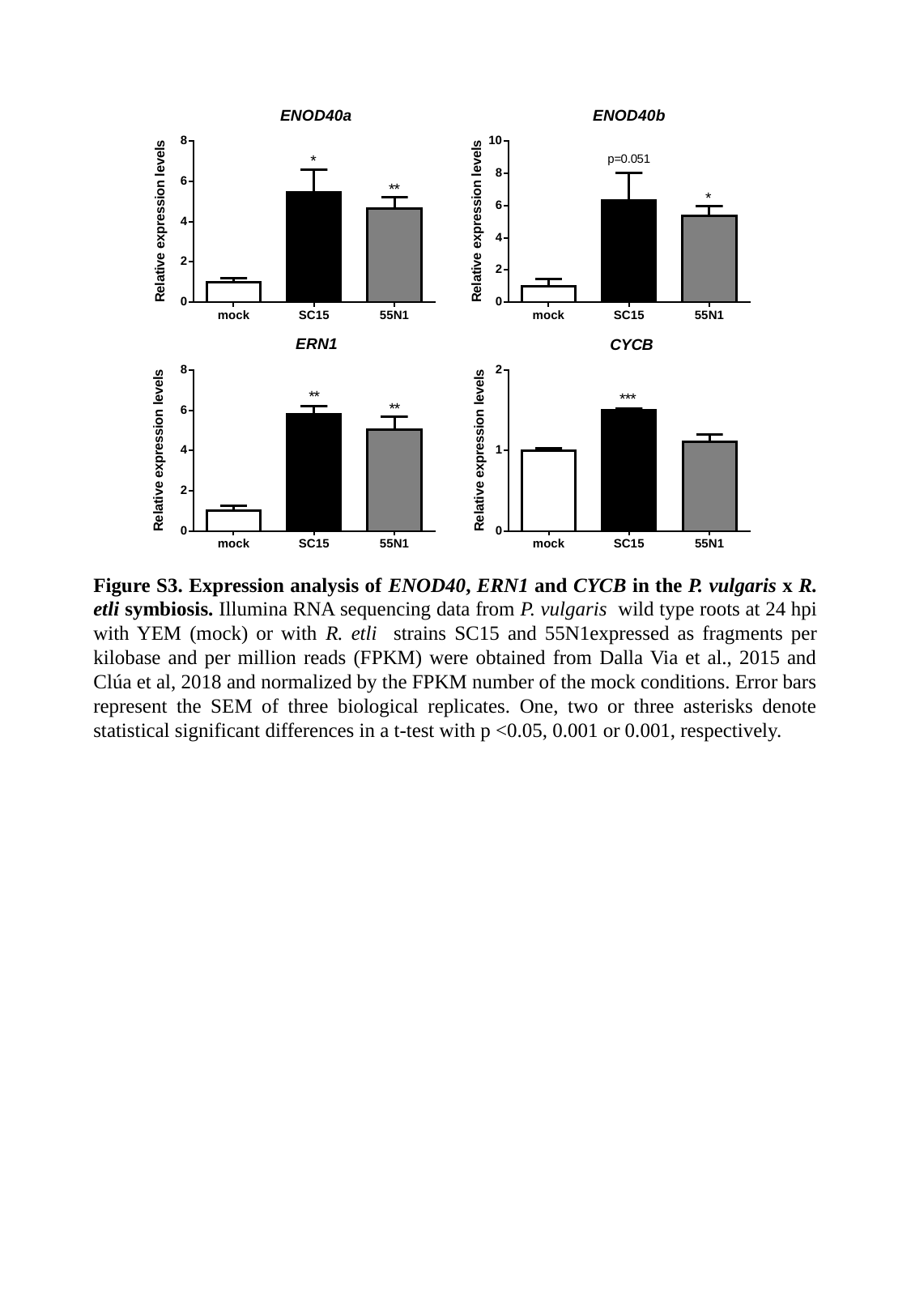

Figure S3. Expression analysis of ENOD40, ERN1 and CYCB in the P. vulgaris x R. etli symbiosis. Illumina RNA sequencing data from P. vulgaris wild type roots at 24 hpi with YEM (mock) or with R. etli strains SC15 and 55N1expressed as fragments per kilobase and per million reads (FPKM) were obtained from Dalla Via et al., 2015 and Clúa et al, 2018 and normalized by the FPKM number of the mock conditions. Error bars represent the SEM of three biological replicates. One, two or three asterisks denote statistical significant differences in a t-test with p <0.05, 0.001 or 0.001, respectively.

## Slide 4
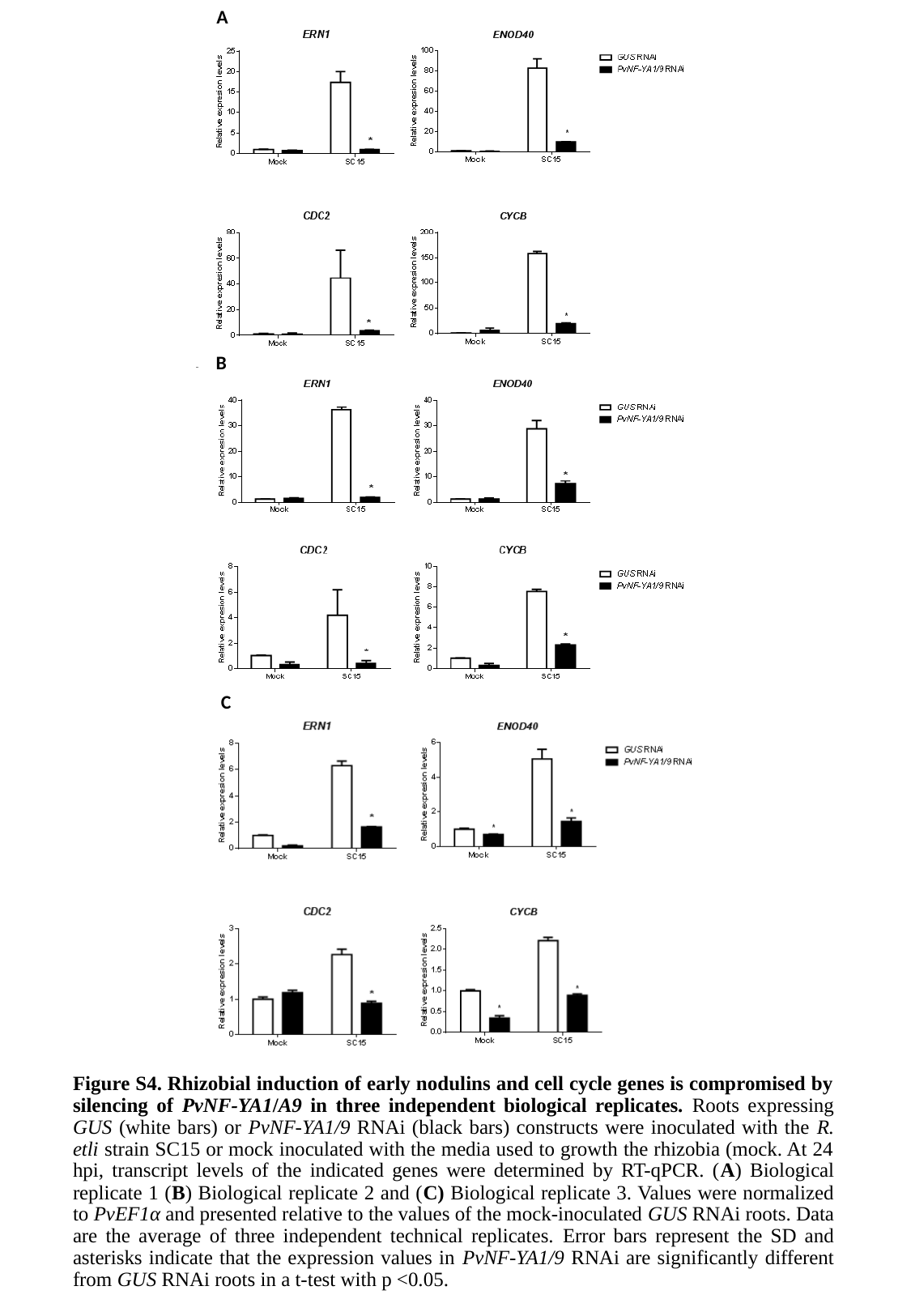

#
Figure S4. Rhizobial induction of early nodulins and cell cycle genes is compromised by silencing of PvNF-YA1/A9 in three independent biological replicates. Roots expressing GUS (white bars) or PvNF-YA1/9 RNAi (black bars) constructs were inoculated with the R. etli strain SC15 or mock inoculated with the media used to growth the rhizobia (mock. At 24 hpi, transcript levels of the indicated genes were determined by RT-qPCR. (A) Biological replicate 1 (B) Biological replicate 2 and (C) Biological replicate 3. Values were normalized to PvEF1α and presented relative to the values of the mock-inoculated GUS RNAi roots. Data are the average of three independent technical replicates. Error bars represent the SD and asterisks indicate that the expression values in PvNF-YA1/9 RNAi are significantly different from GUS RNAi roots in a t-test with p <0.05.

## Slide 5
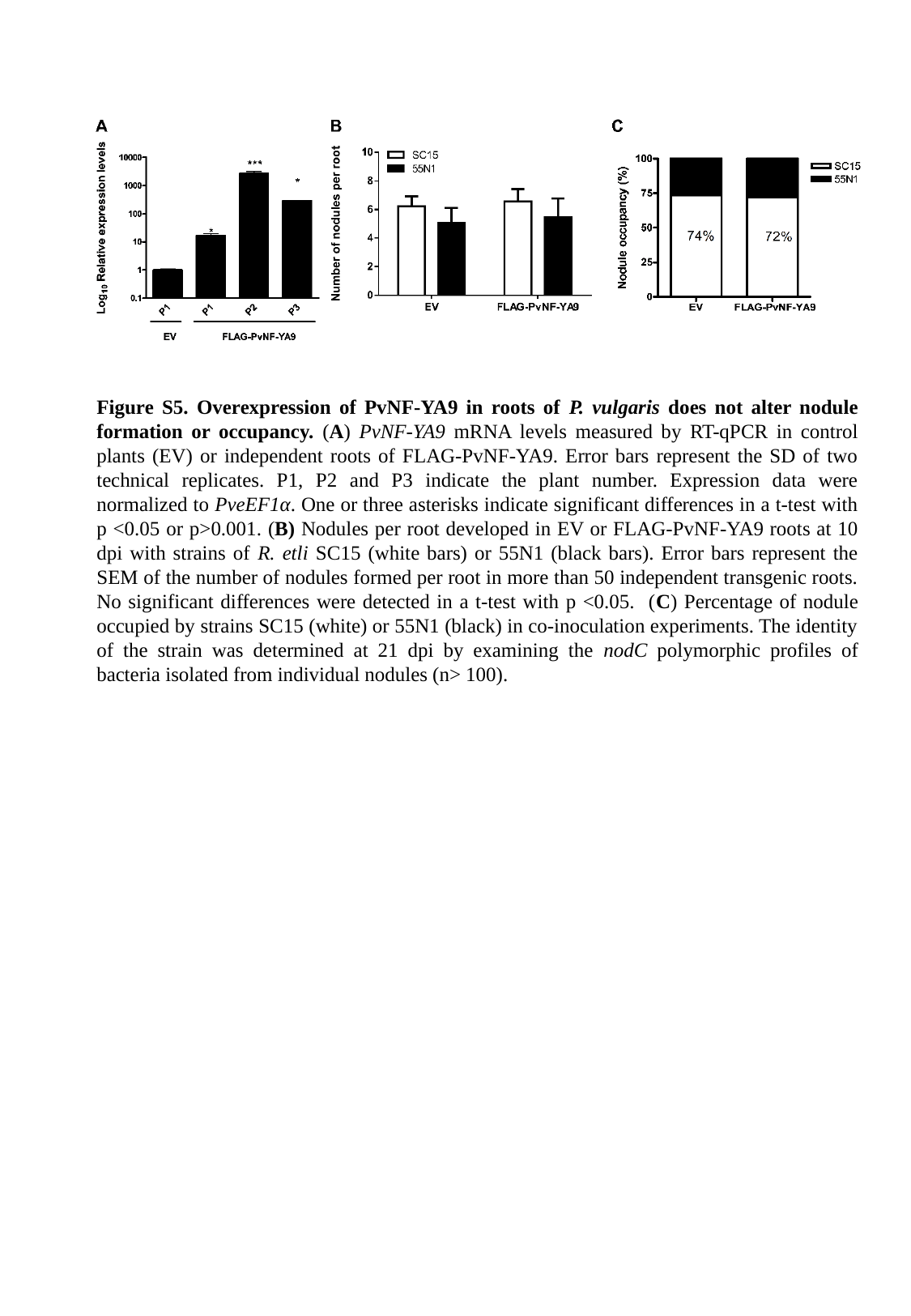

Figure S5. Overexpression of PvNF-YA9 in roots of P. vulgaris does not alter nodule formation or occupancy. (A) PvNF-YA9 mRNA levels measured by RT-qPCR in control plants (EV) or independent roots of FLAG-PvNF-YA9. Error bars represent the SD of two technical replicates. P1, P2 and P3 indicate the plant number. Expression data were normalized to PveEF1α. One or three asterisks indicate significant differences in a t-test with p <0.05 or p>0.001. (B) Nodules per root developed in EV or FLAG-PvNF-YA9 roots at 10 dpi with strains of R. etli SC15 (white bars) or 55N1 (black bars). Error bars represent the SEM of the number of nodules formed per root in more than 50 independent transgenic roots. No significant differences were detected in a t-test with p <0.05. (C) Percentage of nodule occupied by strains SC15 (white) or 55N1 (black) in co-inoculation experiments. The identity of the strain was determined at 21 dpi by examining the nodC polymorphic profiles of bacteria isolated from individual nodules (n> 100).

## Slide 6
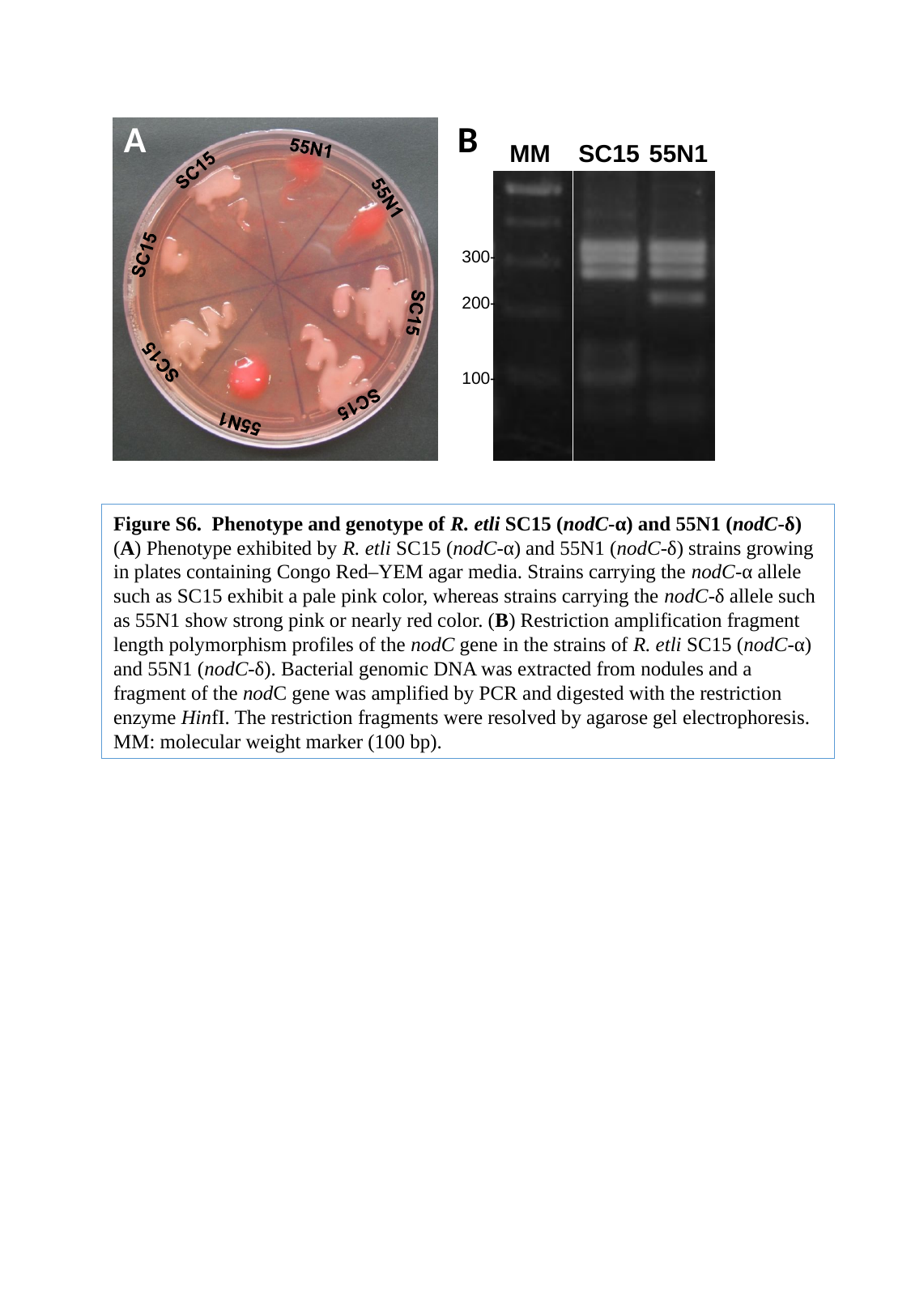

Figure S6. Phenotype and genotype of R. etli SC15 (nodC-α) and 55N1 (nodC-δ) (A) Phenotype exhibited by R. etli SC15 (nodC-α) and 55N1 (nodC-δ) strains growing in plates containing Congo Red–YEM agar media. Strains carrying the nodC-α allele such as SC15 exhibit a pale pink color, whereas strains carrying the nodC-δ allele such as 55N1 show strong pink or nearly red color. (B) Restriction amplification fragment length polymorphism profiles of the nodC gene in the strains of R. etli SC15 (nodC-α) and 55N1 (nodC-δ). Bacterial genomic DNA was extracted from nodules and a fragment of the nodC gene was amplified by PCR and digested with the restriction enzyme HinfI. The restriction fragments were resolved by agarose gel electrophoresis. MM: molecular weight marker (100 bp).
